# A Meta-Analysis of Zilpaterol and Ractopamine Effects on Feedlot Performance, Carcass Traits and Shear Strength of Meat in Cattle

**DOI:** 10.1371/journal.pone.0115904

**Published:** 2014-12-30

**Authors:** Ian J. Lean, John M. Thompson, Frank R. Dunshea

**Affiliations:** 1 SBS*cibus*, Camden, New South Wales, Australia; 2 Environmental and Rural Science, University of New England, Armidale, New South Wales, Australia; 3 School of Land and Environment, University of Melbourne, Parkville, Victoria, Australia; Auburn University, United States of America

## Abstract

This study is a meta-analysis of the effects of the beta-agonists zilpaterol hydrochloride (ZH) and ractopamine hydrochloride (RAC) on feedlot performance, carcase characteristics of cattle and Warner Bratzler shear force (WBSF) of muscles. It was conducted to evaluate the effect of the use of these agents on beef production and meat quality and to provide data that would be useful in considerations on the effect of these agents on meat quality in Meat Standards Australia evaluations. We conducted a comprehensive literature search and study assessment using PubMed, Google Scholar, ScienceDirect, Scirus, and CAB and identification of other studies from reference lists in papers and searches. Searches were based on the key words: zilpaterol, zilmax, ractopamine, optaflexx, cattle and beef. Studies from theses obtained were included. Data were extracted from more than 50 comparisons for both agents and analysed using meta-analysis and meta-regression. Both agents markedly increased weight gain, hot carcase weight and longissimus muscle area and increased the efficiency of gain:feed. These effects were particularly large for ZH, however, fat thickness was decreased by ZH, but not RAC. Zilpaterol also markedly increased WBSF by 1.2 standard deviations and more than 0.8 kg, while RAC increased WBSF by 0.43 standard deviations and 0.2 kg. There is evidence in the ZH studies, in particular, of profound re-partitioning of nutrients from fat to protein depots. This work has provided critically needed information on the effects of ZH and RAC on production, efficiency and meat quality.

## Introduction

Dietary additives, including *β*-adrenergic agonists (***β*** **-AA**) are used by the beef industry to enhance the efficiency of gain and modify carcass characteristics and meat quality [1], [2]. Both zilpaterol (ZH) and ractopamine (RAC) bind to β-adrenergic receptors located in the cellular membranes and indirectly lead to decreased lipogenesis (fat synthesis and storage) and increased lipolysis (fat mobilization and hydrolysis) [3], [4],[5],[6]. The magnitude of these changes is influenced by dose and duration of treatment with the *β*-AA, the type of *β*-AA, and the species of animal treated [7], [5], [8]. The *β*-AA also directly influence protein metabolism. Previous studies have shown that RAC improved growth performance [1], [2], with the effects mediated through increased protein synthesis [8] and decreased protein degradation [5], [8], [6].

A review of the meat science literature [9] concluded that if improvements in animal growth were due in part to decreased protein degradation, this would reduce the rate of post-mortem proteolysis in muscle and result in tougher meat. Given that part of the *β*-AA effect is likely to be associated with protein degradation rates it's use may produce tougher meat. However the results on meat quality from individual studies appear equivocal with a range of individual studies varying in the magnitude and statistical significance of the responses [6].

The objectives of this study were to evaluate the effects of *β*-AA on feedlot performance and carcass traits of cattle, using meta-analytic methods. Meta-analysis integrates the results from many studies to provide a more robust estimate of the effects of *β*-AA (both ZH and RAC) on live and carcass traits. We also examined the heterogeneity or variability of responses in order to better target future research projects and resolve other hypotheses raised about the action of the products and gaps in current knowledge. An understanding of factors that give rise to heterogeneity between studies can also lead to more effective treatments or modifications of management.

## Materials and Methods

### Literature search

Our literature searches used PubMed, Google Scholar, ScienceDirect, Scirus, and CAB and identification of other studies from references lists in papers. Searches were based on the following key words: zilpaterol, zilmax, ractopamine, optaflexx, cattle and beef using the terms for the pharmaceutical agent or product brand names separately with the terms cattle and beef. Where possible, studies from theses obtained were also included. A list of published studies on ZH in beef cattle was provided by Meat and Livestock Australia (MLA). The papers were useful in the initial phase of the project, but did not yield additional studies after the study search was completed. In many cases, authors of articles were contacted to provide clarity in regard to results and for additional information.

### Inclusion and exclusion criteria

Studies were included or excluded in this study based on a series of criteria developed by SBScibus and which are discussed in a review of the use of meta-analysis in animal and veterinary science [12]. Briefly, database searches were augmented with hand searches of library resources for relevant papers, books, abstracts, and conference proceedings. We pursued references in papers and contacted authors active in the field. Quality assessment criteria included randomization of study groups, appropriate analysis of data and comparability of treatment groups at entry to each trial. The extracted data were audited by five reviewers.

References [19]–[102] were evaluated for inclusion or exclusion in the study and details of these studies are provided in File S1. Trials were included in the analysis if they met the following criteria: full manuscripts from peer-reviewed journals, published after 1980, that evaluated use of ZH or RAC supplementation in cattle; had a description of randomization processes; reported the dose of β-AA used, animals studied were cattle; the paper contained sufficient data to determine the effect size for production outcomes (e.g., the number of cattle or pens, carcases or cuts of beef in each treatment and control group); a measure of effect amendable to effect size analysis for continuous data (e.g., standardized mean difference, **SMD**); a measure of variance (SE or SD) for each effect estimate or treatment and control comparisons. Studies were rejected that could not be adequately interpreted in regard to origin of muscles, that used non-representative sampling methods, or whose authors did not respond to clarify their approach.

Design criteria included the number of animals/pen, animals/group and pens/group; experimental and analytical unit (animal, pen, carcass, muscle).Experimental details included days on feed, the number of days products were fed, dosage of products fed (per kg DMI or per kg/hd/d), diet and delivery methods of product and withdrawal period (days) of the test products prior to slaughter. Animal details included class of cattle (steers, heifers, bulls or cull cows); vaccination history, use of anthelmintics; other supplements and growth promoting products (hormonal implants); housing and feeding systems.

Output variables extracted for meta-analysis included final body weight (BW, kg), dry matter intake (DMI, kg/hd/day), average daily gain (ADG, kg/hd/day), gross feed efficiency (G:F ratio); hot carcass weight (HCW, kg), dressing percentage (D%, (kg HCW/kg BW)*100), ultimate muscle pH, longissimus muscle area (LMA, cm^2^),USDA muscle colour score (scale 1 to 9), USDA marbling score; 12^th^ rib fat thickness (mm) and Warner-Bratzler shear force (WBSF, kg). The WBSF was predominantly assessed on striploin samples cooked to internal temperature of 70 to 71°C on a belt grill.

In many papers muscle, bone and overall maturity were reported as a proportion within the A maturity category, whilst in others it was reported as a continuous variable. As it was not possible to reconcile these units, maturity scores were not included in the current analyses. The ADG, DMI and G:F ratio were reported over the period of supplementation, generally 20–30 days, not over the full feeding period. There was also a lack of detail on sampling methods for sensory samples for a number of ZH and RAC studies and it was considered there were insufficient studies to include this trait in the analyses.

### Statistical analysis

We used Stata (Intercooled Stata v.13, USA) to analyze production and carcass data by standardized mean difference (SMD) which is also called effect size (ES) analysis, in which the difference between treatment and control groups means was standardized using the standard deviations of control and treatment groups. The SMD estimates were made for the fixed effect [10], and random effects [11] models that are reported. If the paper reported separate estimates of measure of variance (SE or SD) for each group, these were recorded as such. Many studies reported a common SE or SD and these estimates were used for both control and treatment groups. We also have provided a random effects weighted mean difference (WMD) between treated and control, with the weighting reflecting the inverse of the variance of the studies included according to non-standard method (Stata 13.0 Statacorp, Tx, USA).

Random effects models were conducted for each production outcome to estimate the effect size, 95% confidence intervals, and statistical significance of SMD. We recognize that there is a clustering effect that results from multiple comparisons to a single control group, but have determined that the variance inflation effect will be minor unless there are very large numbers of repeated comparisons. The statistical methods of meta-analytic procedures that were used in this paper have been previously published [12].

Efforts were made to clearly identify the units of interest used in the studies and to clarify the measures of dispersion reported in papers. If there was some uncertainty, authors were contacted to provide clarification of these measures and a number responded. If there was a lack of clarity in regards to the unit of measure, a more conservative measure was used. Specifically, if muscle characteristics were measured and evaluated as the unit of analysis, but the muscles were obtained from pen fed studies, pen was used in our analyses.

#### Forest plots

The effects of *β*-AA on production performance of beef cattle are displayed in the forest plots, using the estimated SMD of *β*-AA using random effects [11] models. Points to the left of the vertical line represent a reduction in the outcome, whereas points to the right of the line indicate an increase in the outcome variable. Each square represents the mean effect size for that study. The upper and lower limit of the line connected to the square represents the upper and lower 95% confidence interval (CI) for the effect size. Forest plots were only presented for selected output variables.

The weighting of a study is estimated by the inverse of the variance of the effect size. Box sizes are proportional to the inverse variance of the estimates. The size of the square box reflects the relative weighting of the study to the overall effect size estimate with larger squares representing greater weight. Boxes draw attention to the studies with the greatest weight. The grey vertical line represents the mean difference of zero or no effect.

#### Assessment of heterogeneity

Variations among the trial level SMD were assessed using a chi-squared (Q) test of heterogeneity. Heterogeneity in studies reflects underlying differences in clinical diversity of the herds and *β*-AA used, differences in study design and analytical methods and statistical variation around responses. Identifying the presence and sources of the heterogeneity improves understanding of the responses to *β*-AA. We used an α level of 0.10 because of the relatively poor power of the χ^2^ test to detect heterogeneity among small numbers of trials [13]. Heterogeneity of results among the trials was quantified using the *I* ^2^ statistic [14], which quantifies the impact of heterogeneity on a meta-analysis, from mathematical criteria, that are independent of the number of studies and the treatment effect metric. *I* ^2^ is a transformation of the square root of the χ^2^ heterogeneity statistic divided by its degrees of freedom and describes the proportion of total variation in study estimates that is due to heterogeneity. Negative values of *I* ^2^ were assigned a value of zero, consequently the value *I* ^2^ lies between 0 and 100%. An *I* ^2^ value greater than 50% indicates substantial heterogeneity.

#### Meta-regression

Meta-regression analyses were used to explore the source of heterogeneity of response, using the individual SMD for each trial as the outcome and the associated standard error as the measure of variance. Meta-regression can be used to explore sources of heterogeneity even if an initial overall test for heterogeneity is non-significant [14]. This also allows us to quantify the magnitude as a function of the *a priori* defined covariate changing and exploring reasons for heterogeneity (i.e. possible/probable study-level predictors). Meta-regression is also a technique that can formally test whether there is evidence of different effects in different subgroups of trials [15]. Inclusion of multiple covariates in the meta-regressions [15], and the use of the smoothed within-trial variance estimates were used to improve hypothesis testing with regard to the significance levels. The permutation test approach for assessing the statistical significance of meta-regression methods [16] used the programming methods [17], [18] to reduce the risk of type I error.

Meta-regression analysis was conducted by first screening individual variables using a P-value of ≤0.10. All variables with P-value of ≤0.10 were entered into a forward stepwise weighted meta-regression, until all remaining variables were significant at P<0.05. Factors identified a priori as factors that might influence responses to treatment were examined, including the unit of study (pen or animal or cut of meat), initial value of the study variable in the control group in each study (eg DMI of control), duration of feeding in the study (days on feed) and period or duration of treatment with each *β*-AA (eg RAC was fed for 30 days). Data were screened for plausible quadratic relationships for these variables by visual appraisal of univariable scatter plots between the covariate and SMD of each study.

#### Publication bias

We investigated the presence of publication bias using funnel plots which are a simple scatter plot of the intervention effect estimates from individual studies plotted against study precision. The name ‘funnel plot’ arises because precision of the estimated intervention effect increases as the size and precision of a study increases. Effect estimates from small studies will scatter more widely at the bottom of the graph and the spread narrows for larger studies. In the absence of bias the plot should approximately resemble a symmetrical (inverted) funnel. If there is bias, for example because smaller studies without statistically significant effects remain unpublished, this will lead to an asymmetrical appearance of the funnel plot and a gap will be evident in a bottom corner of the graph. In this situation, the effect calculated in a meta-analysis will tend to overestimate the intervention effect. The more pronounced the asymmetry, the more likely it is that the bias will be substantial. Individual studies with large or aberrant results were identified from the forest and funnel plots and evaluated for factors that may have influenced the results. This lead to the exclusion of some studies (see Tables 1 and 2).

**Table 1:** Effects of zilpaterol on feedlot performance and carcase characteristics.

Outcome	Number of studies (n)	Raw mean difference 95% CI	Effect Size and 95% CI	Weighted mean difference and 95% CI	I^2^ estimated hetero-geneity	Significant meta-regression effects
Final Body weight (kg)	31	6.562	0.449	8.150	0.0	
		4.229 to 8.895	0.277 to 0.621	5.627 to 10.674		
Dry Matter Intake (kg/d)	26	−0.160	−0.470	−0.118	0.0	
		−0.243 to −0.077	−0.676 to −0.264	−0.167 to −0.070		
Average Daily Gain (kg/d)	29	0.210	0.884	0.153	29.1	
					(0.0)^1^	
		0.141 to 0.278	0.656 to 1.113	0.111 to 0.194		
Gain:feed (kg/kg ratio)	28	0.024	1.336	0.024	54.1	
		0.017 to 0.031	1.002 to 1.671	0.018 to 0.030		
Hot Carcase Weight (kg)	35	15.383	1.323	15.179	55.9	
		12.937 to 17.829	1.034 to 1.611	13.615 to 16.743		
Ultimate pH	5	0.022	0.102	−0.002	0.0	NA
		−0.033 to 0.077	−0.411 to 0.616	−0.017 to 0.013		
Longissimus muscle area (cm^2^)	35	8.147	2.302	8.006	70.8	Unit (0.022) period (0.165) Multivariate^2^
		7.115 to 9.180	1.898 to 2.705	7.052 to 8.959		
Objective measurement of ‘redness’ (Colour)	18	0.012	0.098	0.03	15.0	
		−0.122 to 0.098	−0.174 to 0.371	−0.004 to 0.066		
Fat thickness at the 12th rib (cm)	35	−0.106	−0.697	−0.11	47.8	Fat of control(0.120) and period (0.186) Multivariate^2^
		−0.139 to −0.073	−0.940 to −0.453	−0.158 to −0.080		
Standardised USDA marbling score	34 (28)*	−23.004	−0.861	−22.947	37.3	Unit (0.030) period (0.050) Multivariate^2^
		−31.245 to −14.763	−1.100 to −0.621	−30.330 to −15.564		
Dressing Percentage (%)	27	1.657	2.205	1.706	71.5	
		1.319 to 1.996	1.669 to 2.741	1.510 to 1.902		
Warner-Bratzler Shear Force (kg)	47	1.022	1.212	0.840	61	Age (0.001) unit (0.001) Multivariate^2^
		0.854 to 1.191	1.024 to 1.401	0.720 to 0.960		

CI – Confidence interval.

1 Sensitivity with removal of Avendano-Reyes et al.

NA – insufficient studies to attempt.

2 Higgins and Thompson (2004) method.

*6 trials removed with different units for Raw mean and weighted mean differences.

**Table 2:** Effects of ractopamine on feedlot performance and carcase characteristics.

Outcome	Number of studies (n)	Raw mean difference 95% CI	Effect Size and 95% CI	Weighted mean difference and 95% CI	I^2^ estimated hetero-geneity%	Significant meta-regression effects <0.05 (P)
Final Body weight (kg)	44	6.476	0.397	7.568	50	Body weight of control (0.01)
		3.200 to 9.752	0.238 to 0.557	5.584 to 9.553		
Dry Matter Intake (kg/d)	48	0.027	0.020	−0.003	26.0	
		−0.116 to 0.170	−0.122 to 0.161	−0.089 to 0.082		
Average Daily Gain (kg/d)	49	0.244	0.76	0.193	54.4	Average Daily Gain control (0.035)
		0.150 to 0.337	0.564 to 0.957	0.149 to 0.237		
Gain:feed (kg/kg ratio)	41	0.019	0.772	0.018	47.8	
		0.012 to 0.026	0.583 to 0.961	0.014 to 0.022		
Hot Carcase Weight (kg)	54	7.376	0.47	6.182	46.5	
		3.475 to 11.277	0.312 to 0.628	4.551 to 7.812		
pH	5	−0.027	−0.326	−0.006	0.0	NA
		−0.079 to 0.024	−0.873 to 0.220	−0.023 to 0.011		
Longissimus muscle area (cm^2^)	60	2.43	0.391	1.844	67.1	
		1.497 to 3.363	0.198 to 0.584	1.172 to 2.517*		
Fat thickness at the 12th rib (cm)	45	0.029	0.005	−0.003	43.1	Day on feed (0.012)
		−0.026 to 0.084	−0.171 to 0.182	−0.035 to 0.028		Decreases smd
USDA marbling score	53	−2.471	−0.108	−5.144	0.8	
		−10.216 to 5.274	−0.213 to −0.002	−9.615 to −0.674		
Dressing Percentage (%)	40	0.503	0.131	0.275	66	
		0.221 to 0.784	0.000 to 0.262	0.110 to 0.440	(0)^2^	
Warner-Bratzler Shear Force (kg)	17	0.305	0.429	0.203	0	
		0.188 to 0.421	0.267 to 0.592	0.122 to 0.284		

CI – Confidence interval.

Estimated mean difference.

NA – insufficient studies to attempt.

Jenning et al and Vogel 3 dropped due to implausible standard errors.

*studies with odd values removed.

## Results and Discussion

### Studies identified and information extracted

A comprehensive literature search using 5 search engines identified relevant peer-reviewed papers, theses and proceedings that were published on ZH and RAC supplementation between 2000 and 2013. For both interventions, details were recorded, but are not provided, on sex of cattle, breed, treatments including vaccinations, parasite control, hormonal implants, feeding regimes, country in which the study was conducted and distance cattle were transported to slaughter.

A total of more than 50 studies on ZH, published between 2000 and 2013, were identified. Of these, 31 papers (with 83 sub-trials or comparisons on different outcomes) met the selection criteria. The required data and information from those studies that met the inclusion criteria were extracted and were tabulated and presented in S1 to S5 Tables in S1 File. The data included information on animal performance, carcass characteristics and meat quality. S6 Table in S1 File provides details of studies that were excluded for various reasons.

Similar search, extraction and reporting methods were used for RAC and a total of 31 papers were identified with 68 sub-trials or comparisons on different outcomes. These trials are presented in S7 to S10 Tables in S1 File. Contact with workers in the field provided valuable detail on studies and allowed re-analysis of some data. Again, three reviewers assessed, extracted and validated the data and 8 papers were excluded (S11 Table in S1 File). There were insufficient studies reporting some variables, especially those reporting sensory traits of meat, for meta-analytical evaluation.

The methods of meta-analysis used in this study have been described and published previously [12], [103], [104]. A critical consideration in this study was to ensure that appropriate estimates of evaluative units (eg pen, animal or carcase) were used for the analyses and that the estimates of standard deviation were also appropriate. Our decisions were influenced by advice received on design and analysis of pen studies that the analyses using SAS would report standard errors of the difference that were appropriate to providing estimates of standard deviation [105] Tempelman, pers com 2013). Studies that did not clearly provide the unit of analysis were evaluated at the highest level of unit identified, ie pen. This may provide a conservative bias in the analysis. Similarly, only random effects models were used, as previous work concluded that when there was uncertainty in the evaluative units caused by clustering of observations the random effects model was appropriate [106].

The effect of reported and assessed evaluative unit was examined by testing of this in meta-regression. Only three outcomes, indicated that the evaluative unit significantly influenced the results of meta-regression, LMA, marbling and WBSF for ZH studies (Table 1). However, in all cases the raw mean difference, an unweighted measure, differed for the outcomes for the pen and individual animal studies, suggesting that there may be a biological reason the differences in effect. In all cases, also, the expected effect of ZH, i.e. increased LMA, lower marbling and increased WBSF was accentuated in the group fed animals. The lack of other significant findings in regard to unit of evaluation in the study overall suggest the possibility that whether cattle had access to individual feeders or were fed in pens influenced the LMA, marbling and WBSF for ZH treated cattle, but had little effect on other output variables. It is possible that such results may reflect the differences between single access to feed and competing in the group-fed situation. Competition can increase stress and agonistic behaviours [107] which may heighten responsiveness to endogenous and exogenous adrenergic stimulation. While effects of group size on feed intake are variable, feed efficiency is generally poorer and a poorer nutritional state is also associated with heightened adrenergic responsiveness [108]. We conclude that the evaluative units were probably correctly identified and, consequently, had little influence on results.

### Zilpaterol

The period of feeding cattle was 143.8±56.1 days and the average period that cattle were exposed to ZH was 26.6±9.0 days. The results of the number of studies analysed, raw mean differences for variables, SMD, weighted mean differences, I^2^ estimated heterogeneity and results of meta-regressions evaluating the effects of ZH are provided in Table 1. These results showed that supplementation of cattle with ZH during the last period of feeding resulted in a substantial impact on live and carcass traits of the animal. The weighted mean differences in BW and ADG between ZH and control groups were substantial being of the order of 0.4 and 0.9 standard deviations, respectively. This effect was equivalent to approximately 8 kg BW and 0.15 kg ADG gain more than the controls. Cattle fed ZH also had a lower DMI of the order of 0.5 standard deviations which was equivalent to approximately 0.1 kg/hd/day. Given that ZH resulted in increased BW and ADG and decreased DMI there was a substantial improvement of 1.4 standard deviations in the G:F ratio which was equivalent to 0.02 improvement in G:F.

The increase in HCW due to ZH supplementation was1.3 standard deviations above controls and equivalent to an extra 15 kg of HCW, almost twice the increase in final BW (Table 1). The disproportionate increases in BW and HCW resulted in an improvement of dressing percentage of 2.2 standard deviations, which was equivalent to an increase of 1.7% in dressing percentage.

ZH acts as a repartitioning agent where there is a substantial redirection of nutrients from fat to protein deposition [6]. The magnitude of the metabolic changes in protein deposition was reflected in the large increase in LMA of 2.3 standard deviations, which was equivalent to an increase of ca. 8 cm^2^ in LMA. This effect was not significantly explained by the difference in HCW between treatment and controls or the HCW of the treatment group. Similarly the reduced fat deposition was reflected in a decrease of 0.9 and 0.7 standard deviations in USDA marbling score and fat depth respectively. These differences were equivalent to a decrease in marbling score of 14 units and a decrease of 1 mm in fat depth. There were few data provided in the papers which examined the physiological and health impacts of this very substantial repartitioning of protein and fat in the treated cattle. It is possible that the profound effect of these agents on repartitioning of nutrients is associated with increased death rates reported for cattle treated with ZH and RAC [110]. Loneragan et al. [109], found that the cumulative risk and incidence rate of death was 75 to 90% greater in cattle fed the βAA compared to contemporaneous controls and that during the exposure period, 40 to 50% of deaths among groups fed the βAA were attributed to administration of the drug.

**Figure 1:**
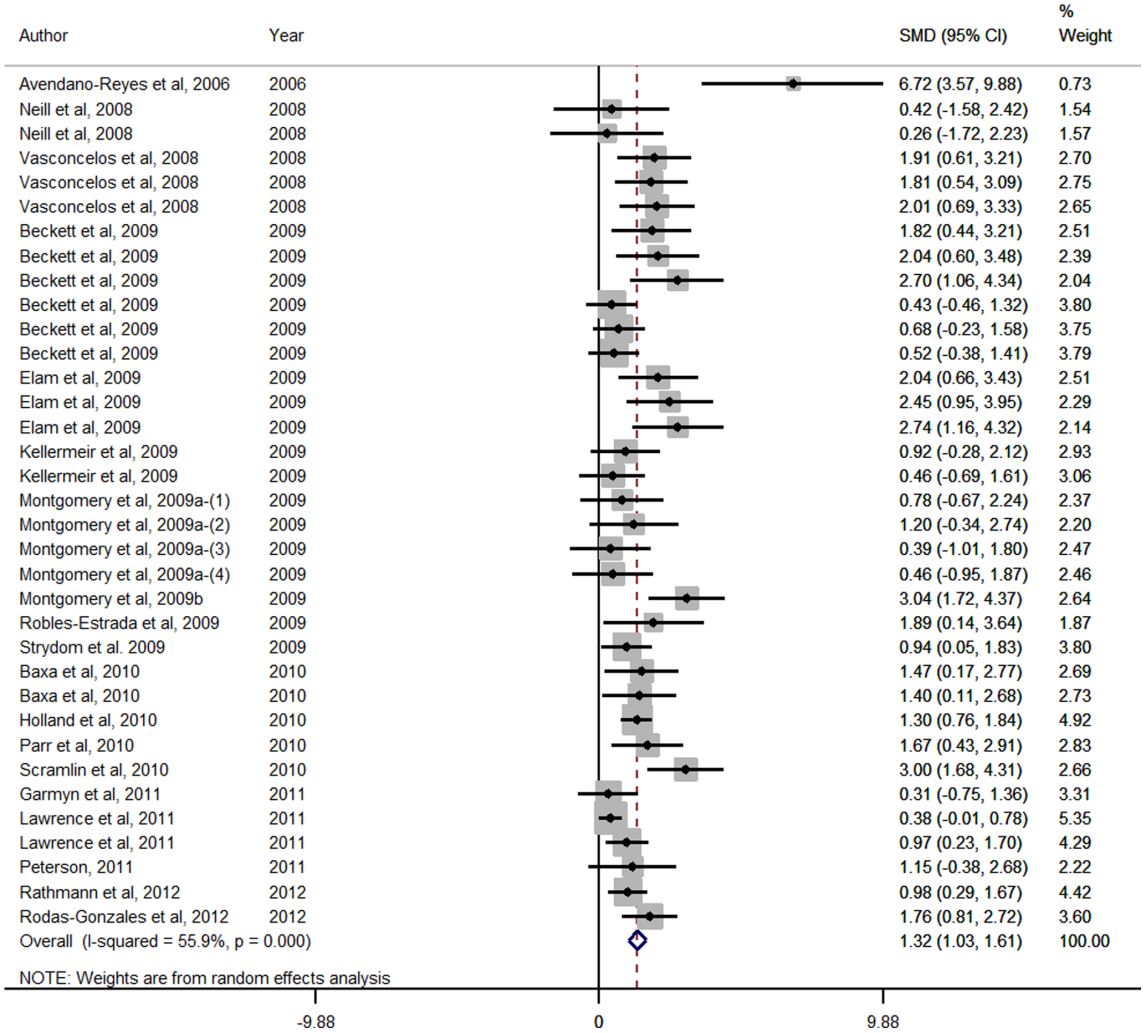
Forest plot of Hot Carcase Weight responses for Zilpaterol studies. A Forest plot of the effect size or standardized mean difference (standardized using the z-statistic) and 95% confidence interval of the effect of zilpaterol treatmenton hot standing carcase weight. The weights that each study contributed are in the right hand column and are indicated by the size of the box. The larger the box, the greater the study contribution to the overall estimate. The solid vertical grey line represents a mean difference of zero or no effect. Points to the left of the line represent a reduction in hot carcase weight, while points to the right of the line indicate an increase. The upper and lower limit of the line connected to the square represents the upper and lower 95% confidence interval for the effect size. The overall pooled effects size and 95% confidence interval is indicated by the diamond at the bottom. This effect was heterogenous as indicated by the I^2^ of 55.9%.

**Figure 2:**
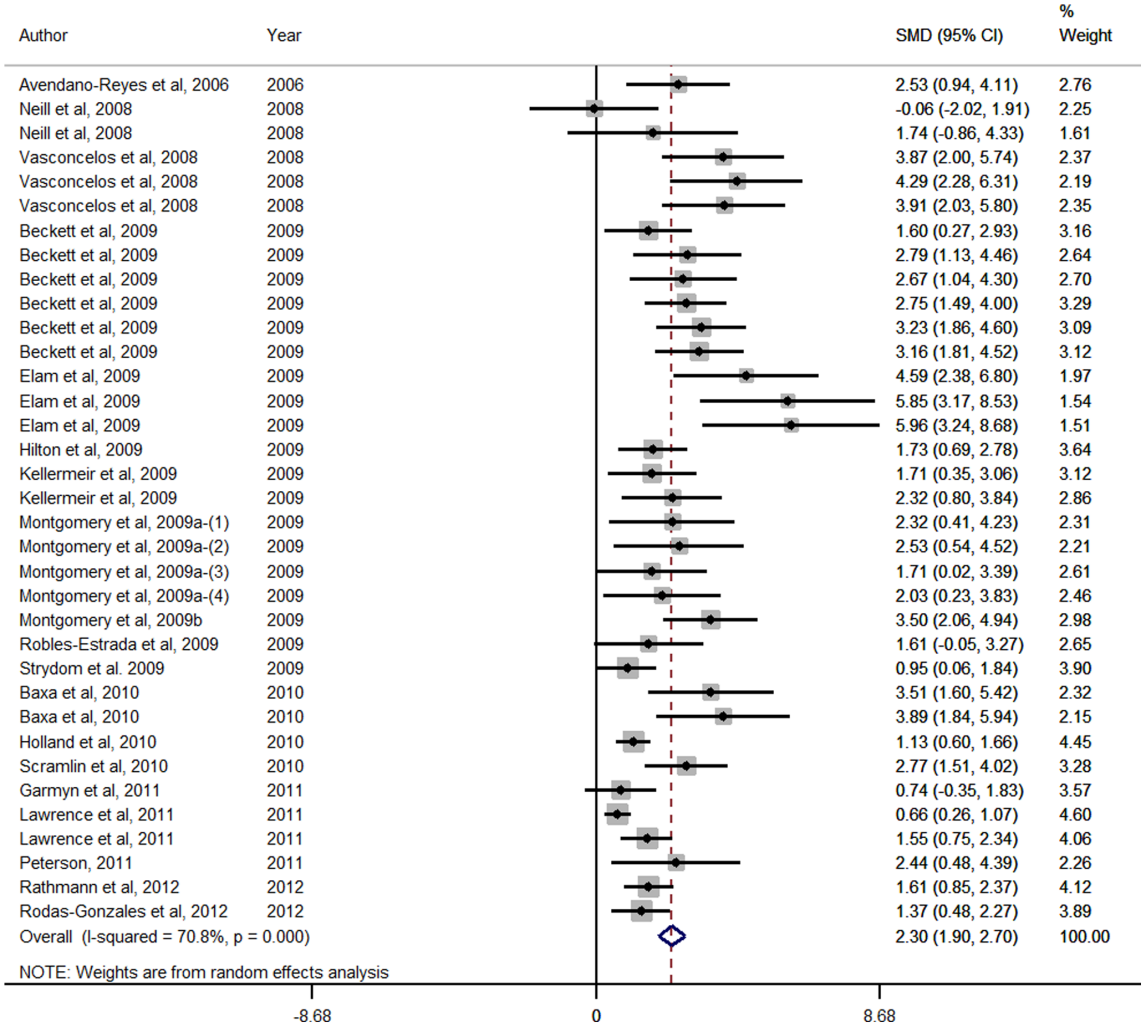
Forest plot of Longissimus muscle area (cm^2^) responses for Zilpaterol studies. A Forest plot of the effect size or standardized mean difference (standardized using the z-statistic) and 95% confidence interval of the effect of zilpaterol treatmenton Longissimus muscle area. The weights that each study contributed are in the right hand column and are indicated by the size of the box. The larger the box, the greater the study contribution to the overall estimate. The solid vertical grey line represents a mean difference of zero or no effect. Points to the left of the line represent a reduction in Longissimus muscle area, while points to the right of the line indicate an increase. The upper and lower limit of the line connected to the square represents the upper and lower 95% confidence interval for the effect size. The overall pooled effects size and 95% confidence interval is indicated by the diamond at the bottom. This effect was heterogenous as indicated by the I^2^ of 70.8%.

**Figure 3:**
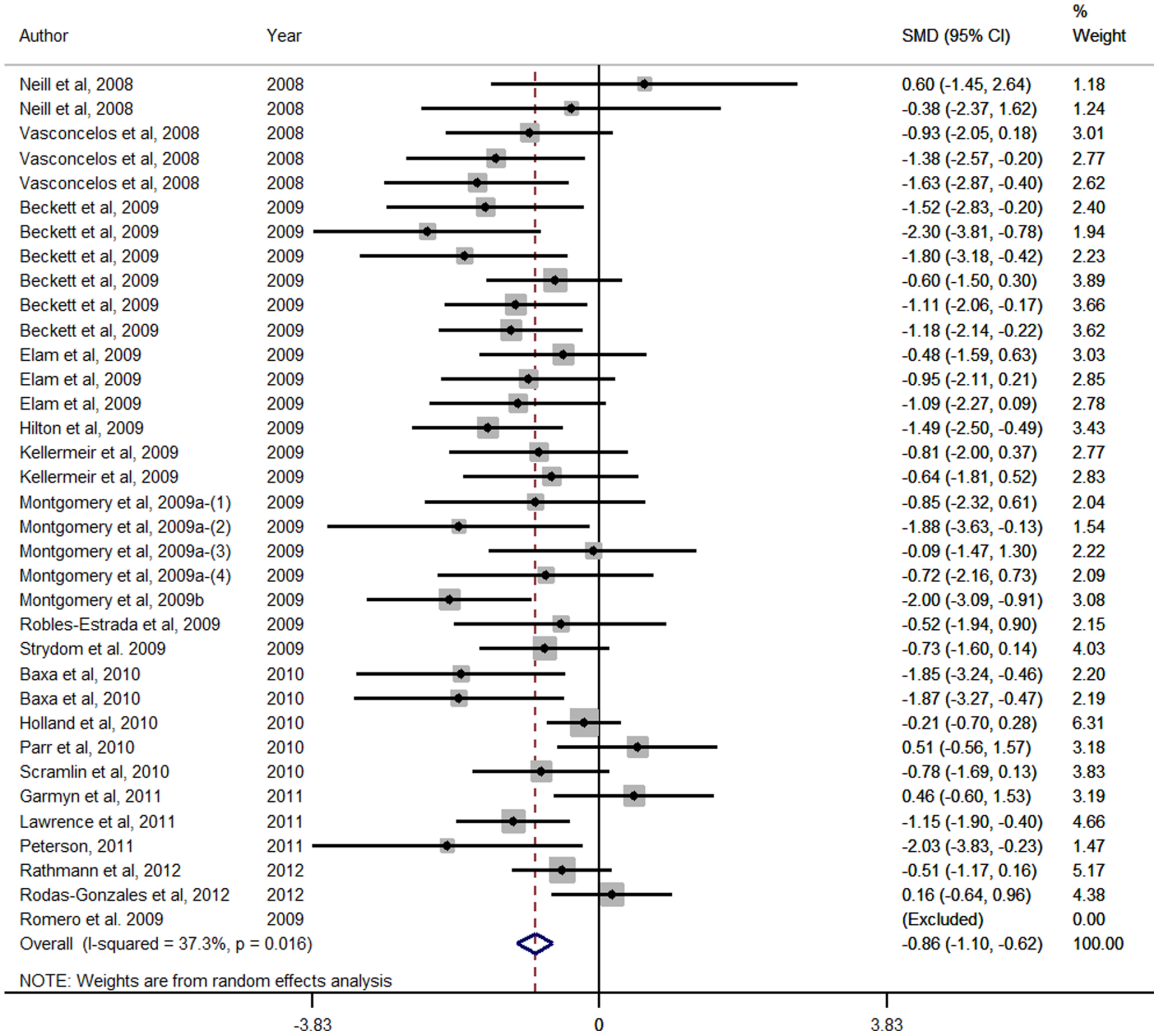
Forest plot of Standardised USDA marbling score responses for Zilpaterol studies. A Forest plot of the effect size or standardized mean difference (standardized using the z-statistic) and 95% confidence interval of the effect of zilpaterol treatmenton USDA Marbling Score. The weights that each study contributed are in the right hand column and are indicated by the size of the box. The larger the box, the greater the study contribution to the overall estimate. The solid vertical grey line represents a mean difference of zero or no effect. Points to the left of the line represent a reduction in Standardised USDA marbling score, while points to the right of the line indicate an increase. The upper and lower limit of the line connected to the square represents the upper and lower 95% confidence interval for the effect size. The overall pooled effects size and 95% confidence interval is indicated by the diamond at the bottom. This effect was relatively homogenous as indicated by the I^2^ of 37.3%.

**Figure 4:**
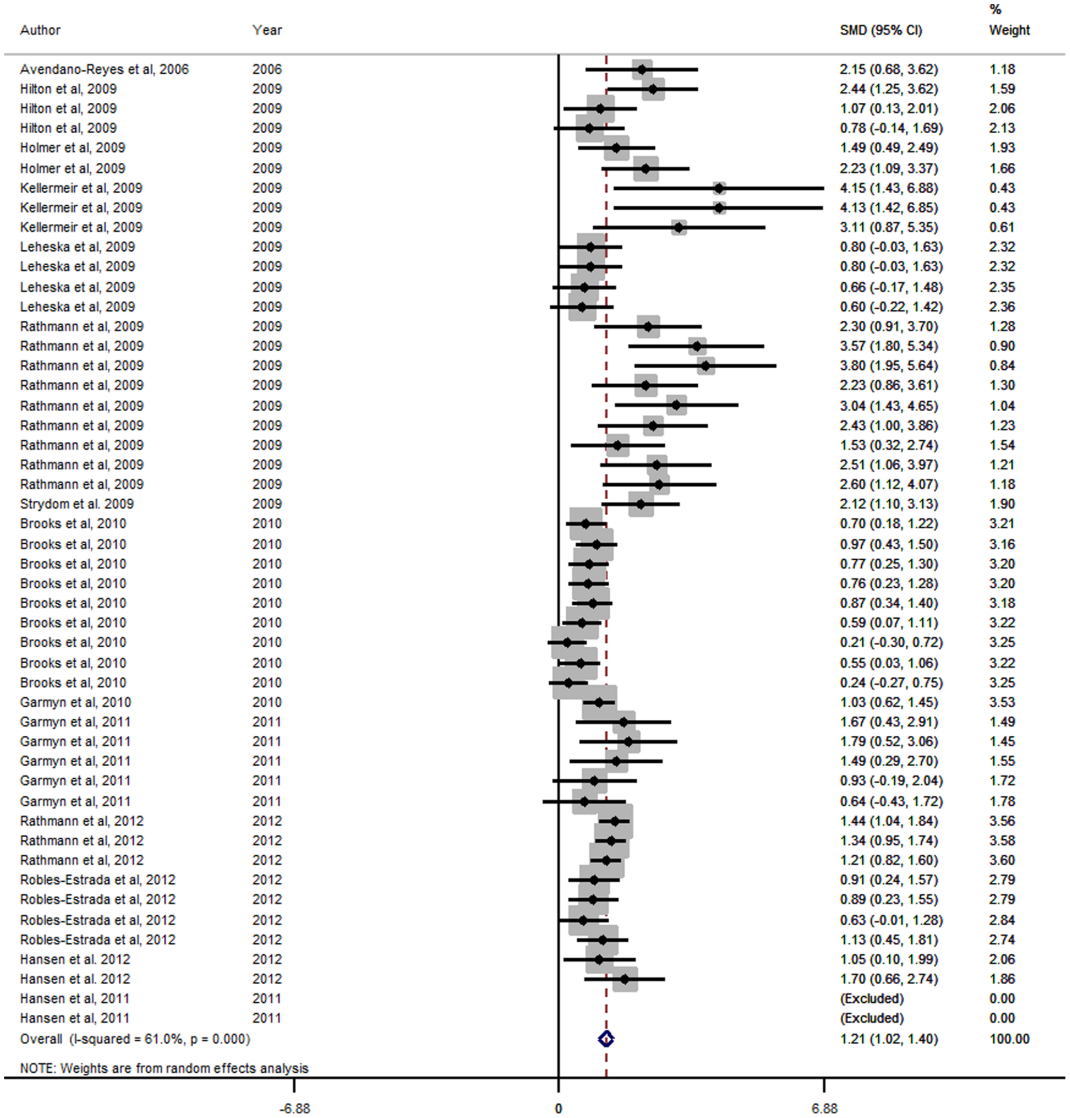
Forest plot of Warner-Bratzler Shear Force responses for Zilpaterol studies. A Forest plot of the effect size or standardized mean difference (standardized using the z-statistic) and 95% confidence interval of the effect of zilpaterol treatmenton Warner-Bratzler Shear Force. The weights that each study contributed are in the right hand column and are indicated by the size of the box. The larger the box, the greater the study contribution to the overall estimate. The solid vertical grey line represents a mean difference of zero or no effect. Points to the left of the line represent a reduction in Warner-Bratzler Shear Force, while points to the right of the line indicate an increase. The upper and lower limit of the line connected to the square represents the upper and lower 95% confidence interval for the effect size. The overall pooled effects size and 95% confidence interval is indicated by the diamond at the bottom. This effect was heterogenous as indicated by the I^2^ of 61.0%.

Forest Plots for HCW, LMA, marbling score and WBSF variables are presented in Figs. 1, 2, 3 and 4 respectively. These Forest Plots demonstrate the consistency of the ZH responses in key commercial carcass and quality traits. Funnel plots were produced, but are not presented. These plots did not show any marked asymmetry with the possible exception of gain:feed. Given the lack of evidence of missing studies in other variables derived from many of the same studies, we conclude that there is little or no evidence of publication bias.

**Figure 5:**
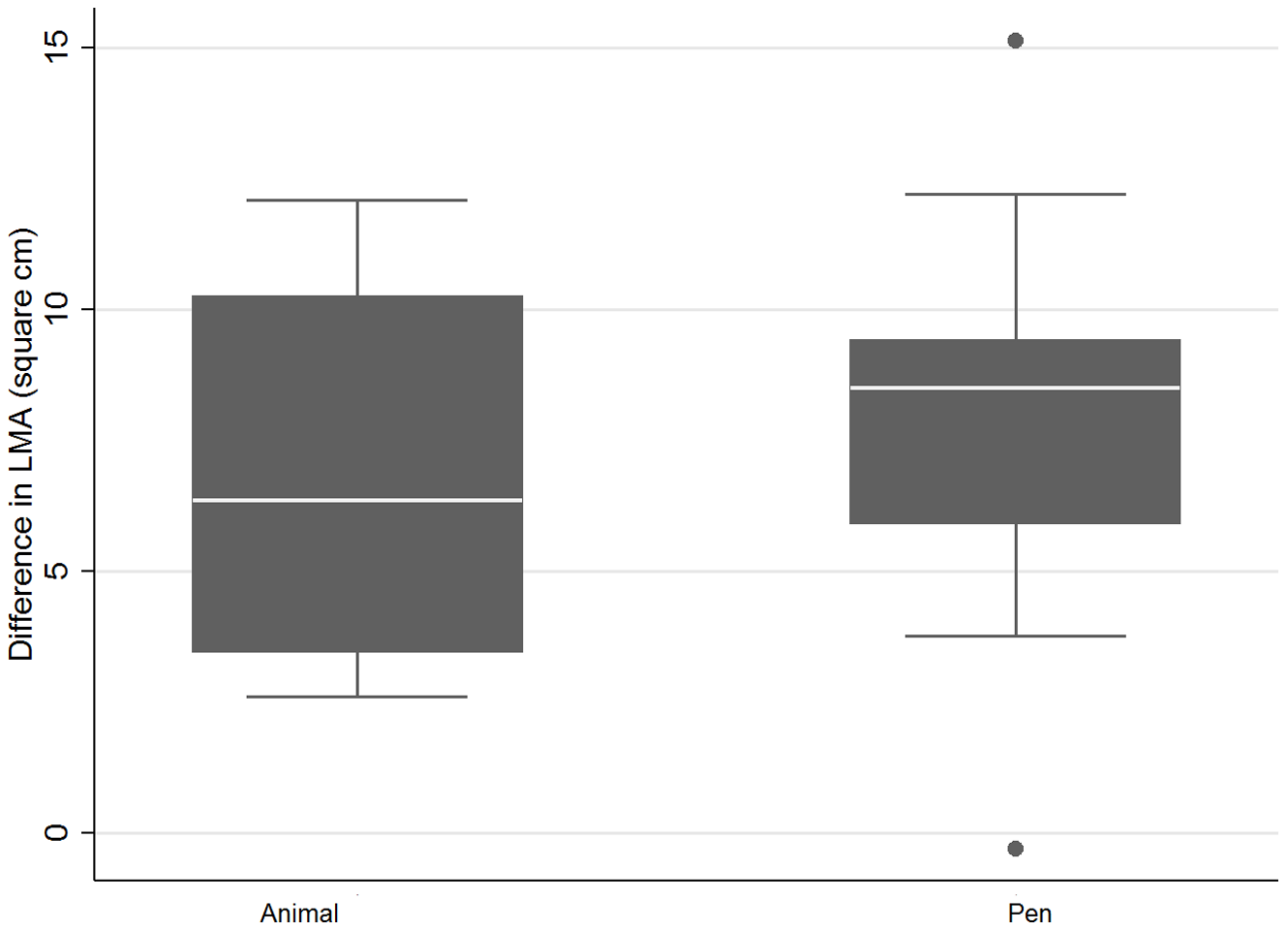
Raw mean differences for studies using animal or pen as the unit of interest for the effect of Zilpaterol on Longissimus muscle area (cm^2^). The dots in the Figures represent studies that are outliers.

Table 1 also reported an estimate of the heterogeneity for the output traits. Most traits had small estimates of heterogeneity with the exception of LMA and D%. Investigation of the sources of this heterogeneity using meta-regression identified that significant heterogeneity in LMA was attributable to the period of feeding of ZH, further emphasising the causal basis of the effect. However, the unit of evaluation, in this case animal or pen indicated that the SMD of LMA was greater in the pen fed studies. As shown in Fig. 5 this difference was evident in the raw, unweighted data, showing that the effect was not mediated through a difference in weighting of studies. It is therefore likely that this result and that for marbling in which the unit of measure was also a significant covariate and that similarly had a difference between pen and animal studies in the raw means reflect differences in expression of effect of ZH between pen fed animals and individually fed animals. The period of feeding of ZH was significant in increasing LMA and approached significance in reducing marbling, again indicating the biological significance of treatment. It is possible that the sensitivity to the ZH is increased under the possibly more stressful group fed conditions in which epinephrine levels may be higher.

**Figure 6:**
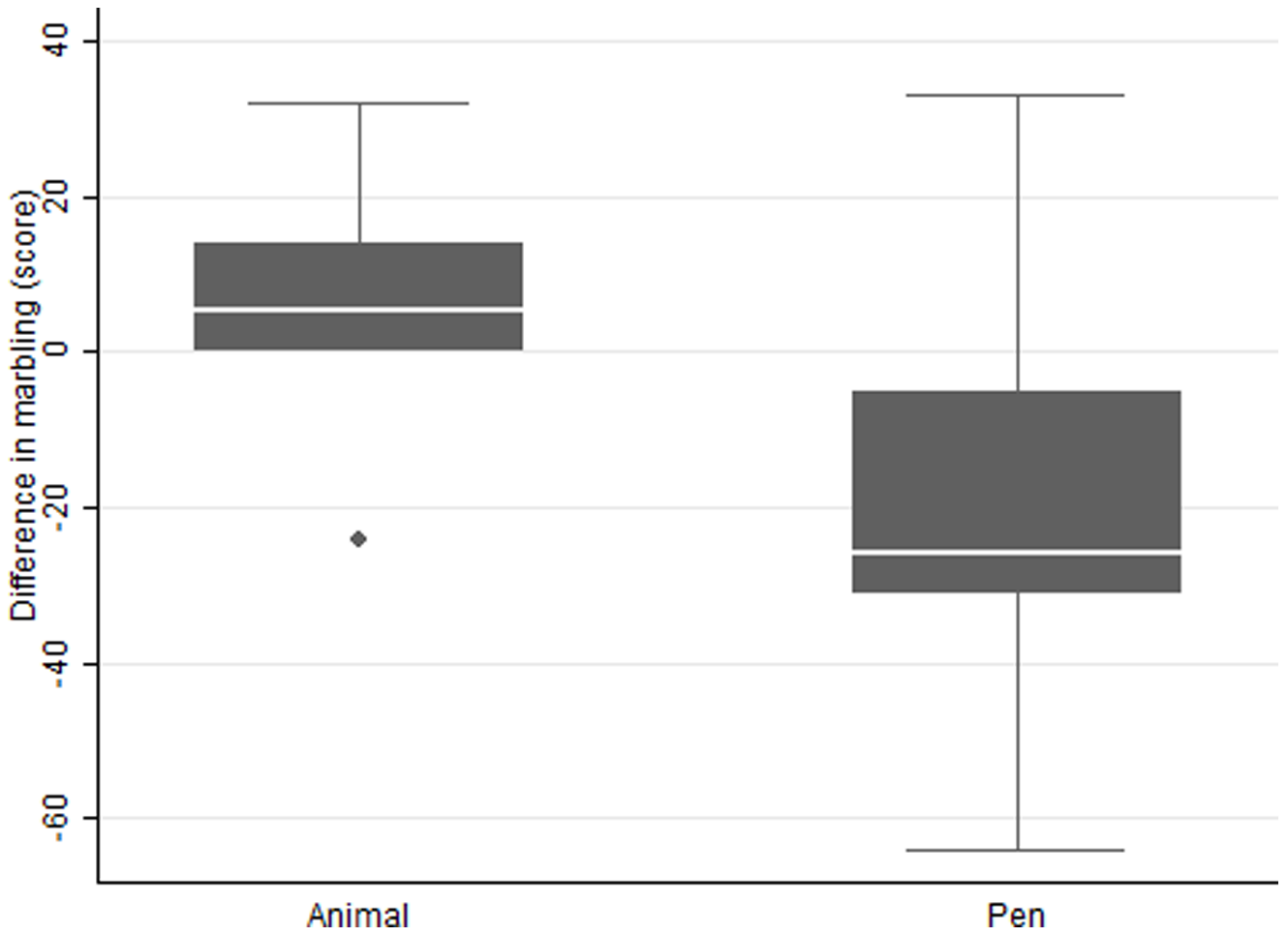
Raw mean differences for studies using animal or pen as the unit of interest for the effect of Zilpaterol on USDA marbling score. The dots in the Figures represent studies that are outliers.

Figs. 5 and 6 show the difference in SMD and raw mean difference for individually fed animals and pen fed groups for LMA and for USDA marbling score, respectively. In studies where animals were individually fed, the response in LMA was smaller than for pen fed groups. In contrast, the reverse was evident for USDA marbling score where individually fed animals showed little response to ZH supplementation compared with group fed animals which showed a large decrease in marbling score. Obviously behavioural changes or competition effects between animals are driving these effects. The authors are not aware of these effects being reported previously. The effect of feeding system on the magnitude of the responses highlights the need to evaluate products under the commercial conditions in which they will be used.

**Figure 7:**
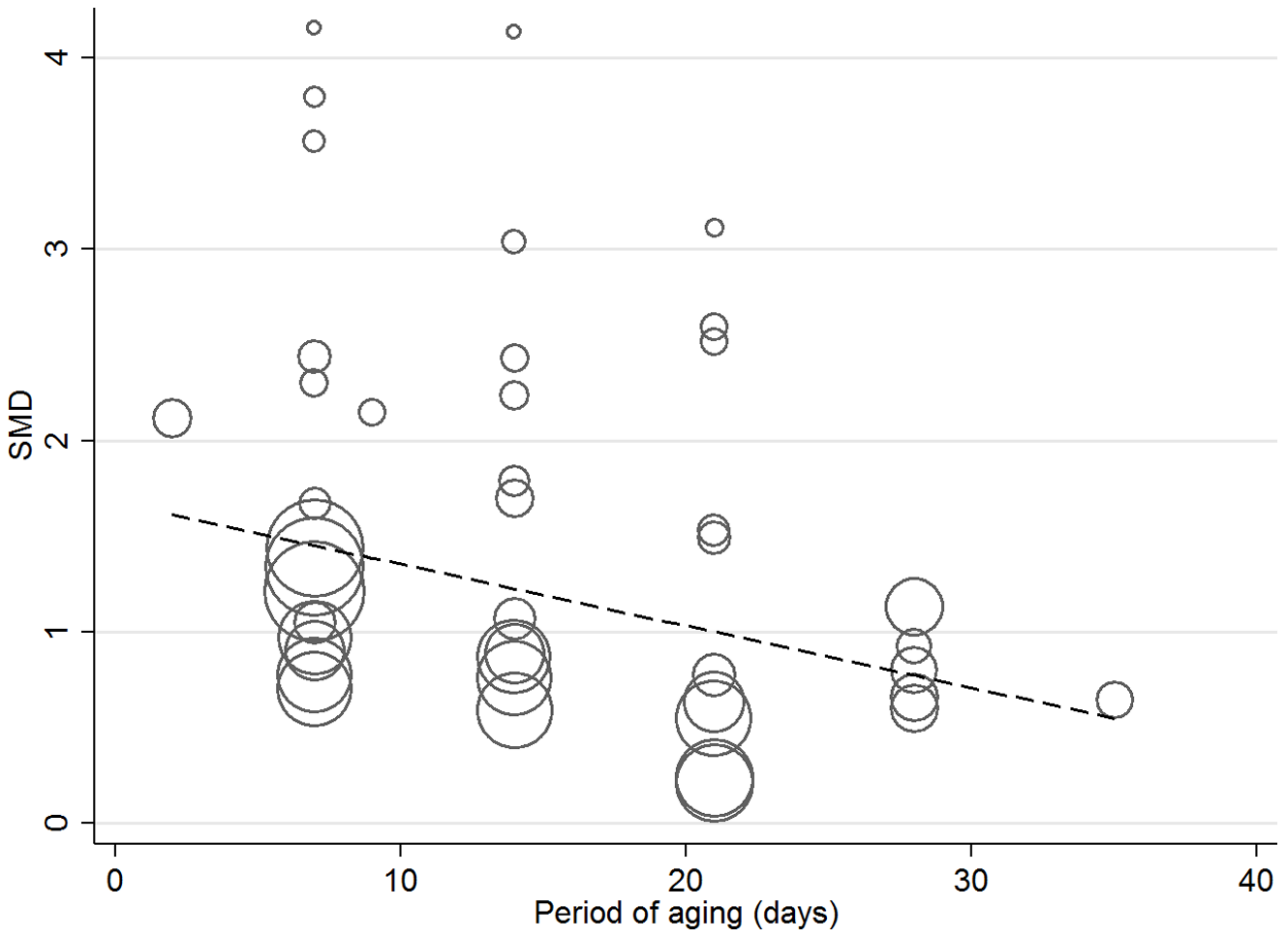
Meta-regression of the effect of aging of steak (days) on the standardised mean difference of studies examining Zilpaterol and Warner-Bratzler Shear Force. The regression is weighted by the effect size of studies which are indicated by the size of the marker. The larger the marker, the greater the effect size of the study.

The effect of ZH on WBSF was large being increased by SMD 1.2 and a weighted mean difference increase of more than 0.8 kg in WBSF (Table 1). There were several significant interactions identified by meta-regression. The magnitude of the ZH effect on WBSF decreased with days that the meat was aged. (Fig. 7).If dietary supplements or implants lower protein degradation rates in the live animal, these are likely to reduce post-mortem ageing rates of the carcase and produce tougher meat [9]. As the improvement in tenderness is exponential, it follows that as meat ages differences between ZH supplemented and control carcasses will decrease.

**Figure 8:**
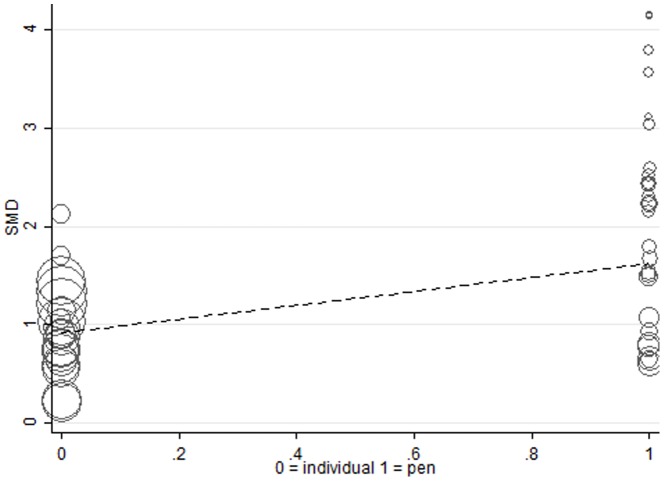
Meta-regression of the effect of individually fed or pen fed on the standardised mean difference of studies examining Zilpaterol and Warner-Bratzler Shear Force. The regression is weighted by the effect size of studies which are indicated by the size of the marker. The larger the marker, the greater the effect size of the study.

The type of study design, that is, whether the study was conducted at the animal level or the pen level also influenced WBSF. Fig. 8 showed an increase in the magnitude of the ZH effect for experiments where pen was the experimental unit. The implications drawn about the unit of evaluation for WBSF are difficult because animal was used as the unit of evaluation in some studies where carcases were randomly selected within a group of cattle fed in pens. However, all studies were in the same direction, indicating an increase in WBSF. The heterogeneity identified, however, is only in degree of increase of WBSF, reflecting quite substantial differences in study design among the studies displayed in Fig. 8.

### Ractopamine

Cattle were fed on average 114.0±44.8 days in the lot and the average period that cattle were exposed to RAC was 30.8±5.3 days. The results of the studies, which pertain to the RAC exposure period, include the raw mean differences for variables, SMD, weighted mean differences, I^2^ estimated heterogeneity and results of meta-regressions evaluating the effects of RAC and are provided in Table 2. Animals supplemented with RAC showed an increase of 0.4 and 0.8 standard deviations in BW and ADG, respectively, which were equivalent to about 8 kg and 0.19 kg/day. In contrast to ZH, the increase in weight gain with RAC was not mediated through increased DMI. The point direction was very close to a zero effect and the overall data showed a marked variation around the mean with some studies showing significant increases or significant decrease in DMI. There was no evidence in this case of missing data from the funnel plot and none of the covariates were significant in explaining the effects of the DMI.

**Figure 9:**
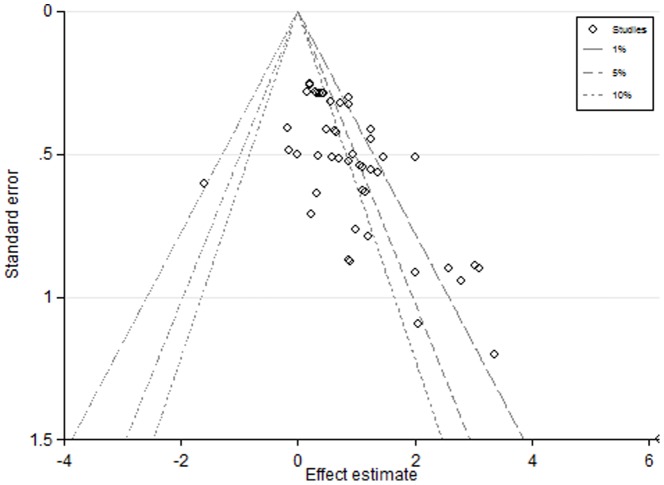
A contour enhanced funnel plot of the effects of RAC on Average Daily Gain.

While the increase in ADG during the period of supplementation was substantial, some caution should be expressed in regard to this result as there is some evidence of missing data on the funnel plot (Fig. 9). It appears that small negative studies may not be present in this particular data set. The covariates examined did not explain any of the variation in effect size. The gain:feed was significantly improved and quite similar in effect size to the response in regard to the ADG. This is unsurprising given the lack of significant increase in DMI observed. It clearly indicates that there are efficiency gains with treatment and the funnel plot was quite symmetric. It is possible, therefore that the funnel plots in the ADG data may be an artefact, reflecting the large number of investigations, of funnel plots in this study. Again none of the sources of variation investigated by meta-regression significantly influenced the gain to feed efficiency.

**Figure 10:**
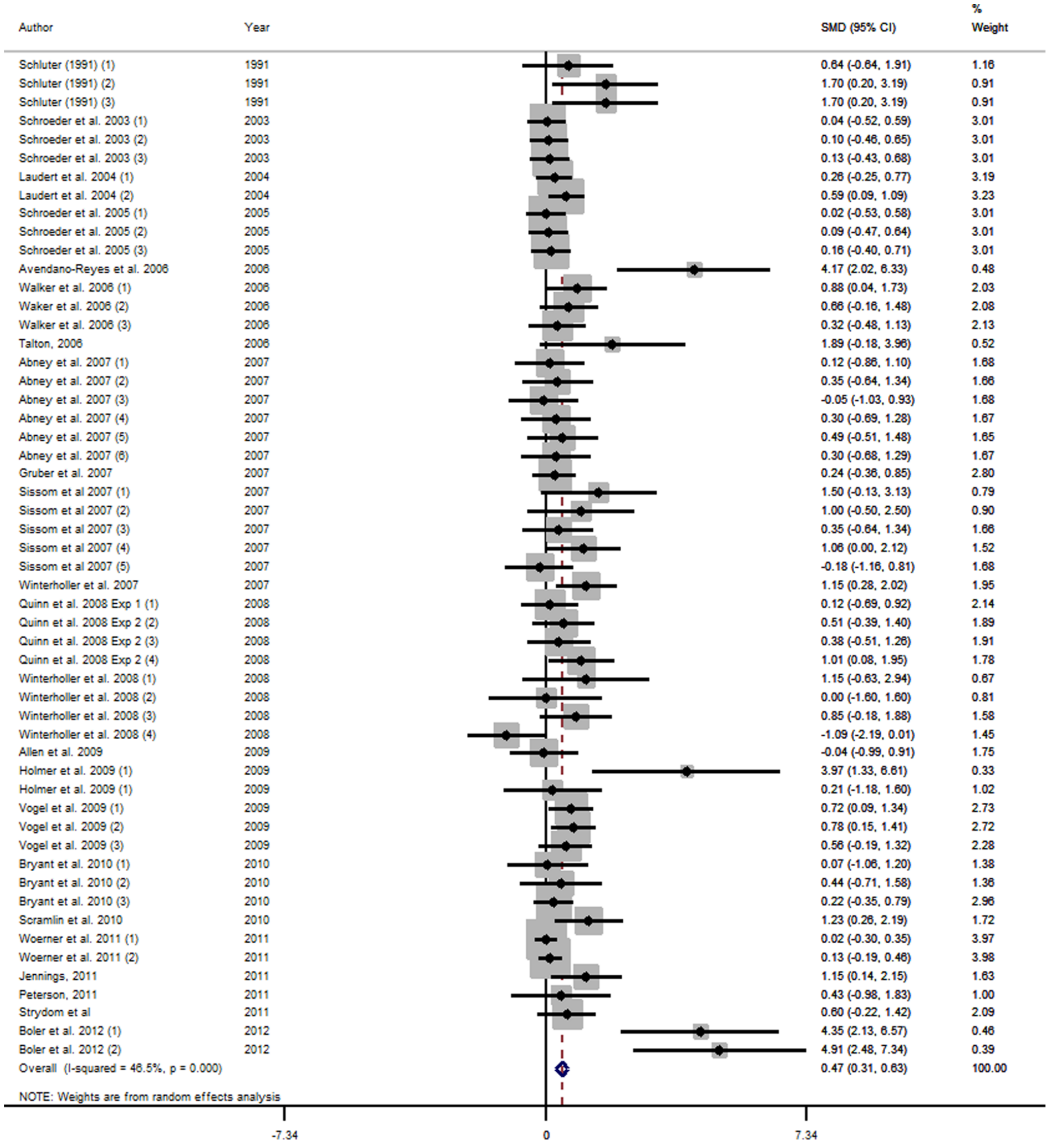
Forest plot of hot carcass weight for ractopamine studies. A Forest plot of the effect size or standardized mean difference (standardized using the z-statistic) and 95% confidence interval of the effect of ractopamine treatmenton hot standing carcase weight. The weights that each study contributed are in the right hand column and are indicated by the size of the box. The larger the box, the greater the study contribution to the overall estimate. The solid vertical grey line represents a mean difference of zero or no effect. Points to the left of the line represent a reduction in hot carcass weight, while points to the right of the line indicate an increase. The upper and lower limit of the line connected to the square represents the upper and lower 95% confidence interval for the effect size. The overall pooled effects size and 95% confidence interval is indicated by the diamond at the bottom. This effect was heterogenous as indicated by the I^2^ of 46.5%.

**Figure 11:**
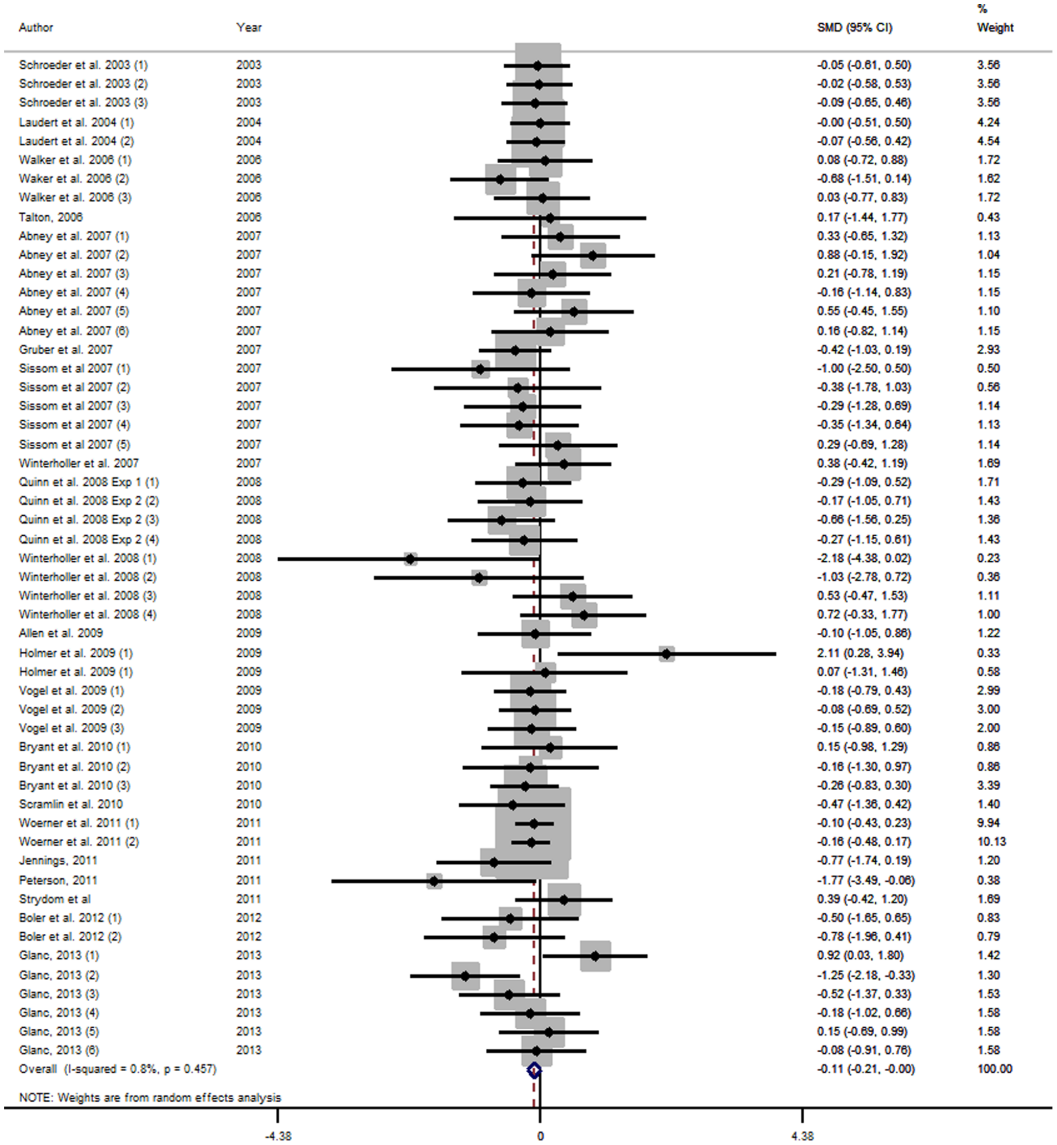
Forest plot of Standardised USDA marbling score for Ractopamine studies. A Forest plot of the effect size or standardized mean difference (standardized using the z-statistic) and 95% confidence interval of the effect of ractopamine treatmenton USDA marbling score. The weights that each study contributed are in the right hand column and are indicated by the size of the box. The larger the box, the greater the study contribution to the overall estimate. The solid vertical grey line represents a mean difference of zero or no effect. Points to the left of the line represent a reduction in Standardised USDA marbling score, while points to the right of the line indicate an increase. The upper and lower limit of the line connected to the square represents the upper and lower 95% confidence interval for the effect size. The overall pooled effects size and 95% confidence interval is indicated by the diamond at the bottom. This effect was homogenous as indicated by the I^2^ of 0.8%.

**Figure 12:**
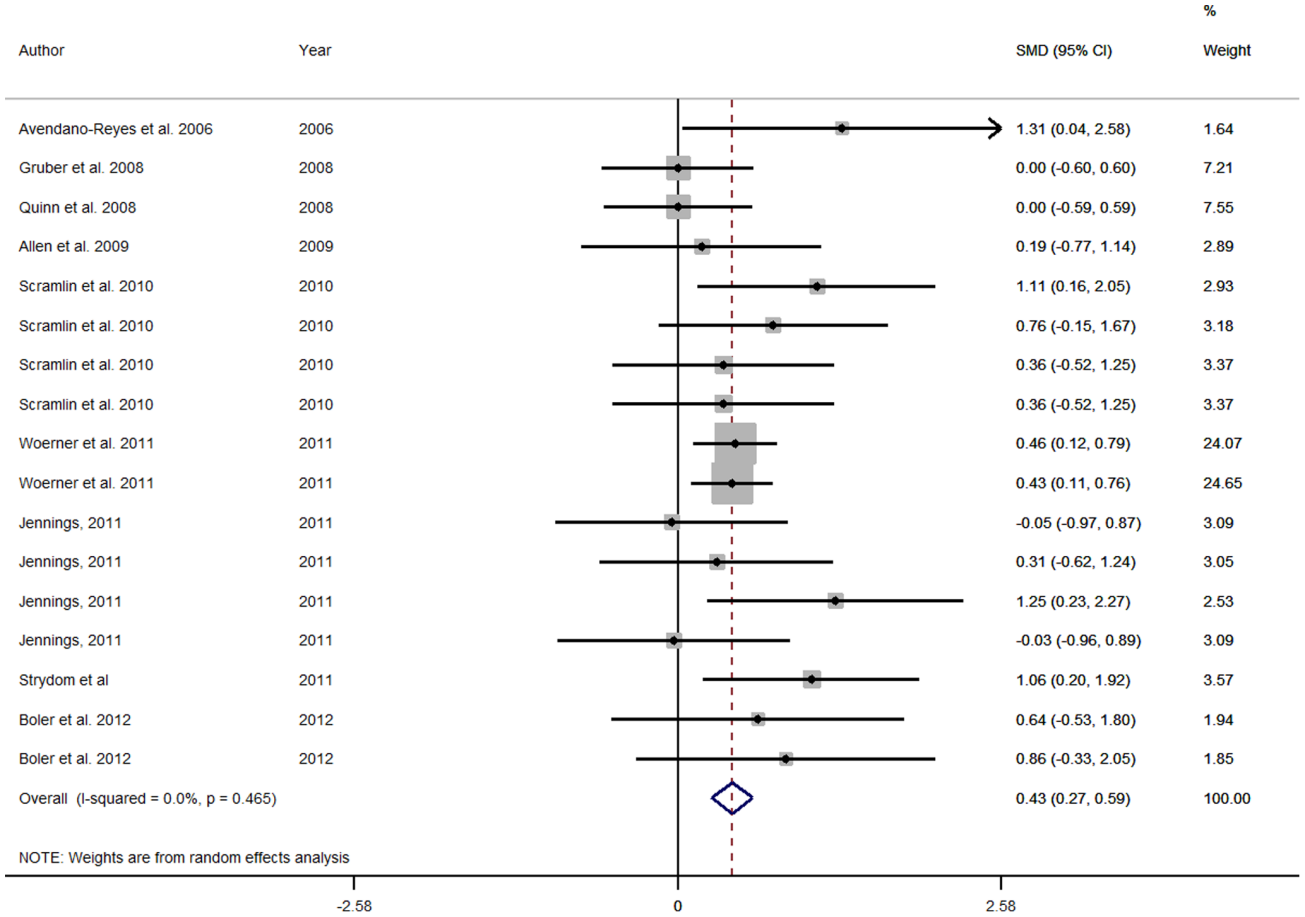
Forestplot of Warner-Bratzler Shear Force for ractopamine studies. A Forest plot of the effect size or standardized mean difference (standardized using the z-statistic) and 95% confidence interval of the effect of ractopamine treatmenton Warner-Bratzler Shear Force. The weights that each study contributed are in the right hand column and are indicated by the size of the box. The larger the box, the greater the study contribution to the overall estimate. The solid vertical grey line represents a mean difference of zero or no effect. Points to the left of the line represent a reduction in Warner-Bratzler Shear Force, while points to the right of the line indicate an increase. The upper and lower limit of the line connected to the square represents the upper and lower 95% confidence interval for the effect size. The overall pooled effects size and 95% confidence interval is indicated by the diamond at the bottom. This effect was homogenous as indicated by the I^2^ of 0%.

Forest plots for HCW, USDA marbling score and WBSF are presented in Figs. 10, 11 and 12. The Forest Plots for RAC supplementation show that the magnitudes of the RAC responses were generally smaller than those for ZH. Although not presented, funnel plots were calculated and showed little evidence of missing studies and consequent publication bias. However, there was substantial evidence of small study effects with these studies sometimes having quite large effects that differed from the larger studies.

The pH data were sparse and not significant. Evidence of repartitioning of nutrients was present in the carcase data. The SMD for LMA and dressing percentage was higher for the treated cattle and the SMD for marbling was significantly lower for the RAC treated cattle, as was the yield grade. The rib fat thickness was not significantly lower for RAC. These findings are consistent with the physiological action of RAC [5]. These results, in combination indicate that the response was to increase muscle, as indicated by increased LMA and HCW, but to decrease lipid as indicated by the marbling scores. The WBSF was also significantly increased by RAC treatment. The carcase data were less heterogenous than the feedlot data with only the HCW and LMA I^2^ approaching or exceeding 50, respectively.

## Conclusions

These findings support the previously identified physiological roles of the *β*-AA and provide strong evidence for producers and others to examine and consider the effects of ZH and RAC on beef cattle production. A large number of reviews and basic physiological studies have not clearly identified the mechanisms by which the actions of the specific *β*-AA are exerted, however, this study clearly demonstrates that the repartitioning effects are rapid, marked and highly integrated.

Once these results have been critically reviewed by others, they can be immediately applied and used to formulate strategies to make best use of agents that markedly improve the efficiency of production. Registration is being sought for these products in countries including Australia. The adoption of these technologies will necessarily involve a consideration of the benefits in production costs and associated environmental benefits of improved efficiencies of resource use against the effects on shear force.

This work provided information on the effects of ZH and RAC on production, efficiency and meat quality. The meta-analysis provided more precise and robust estimates of the effects of the *β*-AA on efficiency of production and carcase quality measures. We identified, using meta-regression, that the method of feeding cattle may influence responses to ZH and that aging of steaks can reduce the effects of ZH on WBSF.

## Acknowledgments and Funding

We are particularly grateful to Professor Mike Gaylean for provision of raw data. We also sincerely thank Professor Robert Tempelman for discussions on the unit of interest, error estimates and study design. We acknowledge the contributions of the staff at SBScibus to the project.

## Supporting Information

S1 Checklist PRISMA Checklist.
(DOC)

S1 File Contains the following files: **Table S1.** Authors, experimental unit, number of cattle and pens per group, and means for control (Con) and treatment (ZH) groups for final body weight (BW), average daily gain (ADG),dry matter intake (DMI) and Gain/Feed (G/F) ratio. **Table S2.** Authors, number of cattle per group, experimental unit, number of cattle and carcasses per group for analysing carcass characteristics in experiments with control (Con) and treatment (ZH) groups. **Table S3.** Authors and study means for control (Con) and treatment (ZH) groups for hot carcass weight (HCW), longissimus muscle area (LMA), ultimate pH and muscle colour score. **Table S4.** Authors and study means for control (Con) and treatment (ZH) groups for USDA marbling score, fat thickness (mm) and dressing percentage (D%). **Table S5.** Author, experimental unit, days aged, number of animals, number of pens, number of muscles and study means for control (Con) and treatment (ZL) groups for Warner Bratzler shear force (WBSF)and cooking loss percentage (CL%). **Table S6.** Zilpaterol studies that did not meet the selection criteria, had insufficient data or data provided were not appropriate. **Table S7.** Authors, experimental unit, number of cattle and pens per group, and means for control (Con) and ractopamine treatment (RAC) groups for final body weight (BW), average daily gain (ADG), dry matter intake (DMI) and Gain/Feed (G:F) ratio. **Table S8.** Authors, \and means for control (Con) and ractopamine treatment (RAC) groups for hot carcass weight (HCW), longissimus muscle area (LMA) and ultimate muscle pH. **Table S9.** Authors and means for control (Con) and ractopaminetreatment (RAC) groups for USDA marbling score, fat thickness (mm) and dressing percentage (D%). **Table S10.** Authors, experimental unit, days aged, number of animals, number of pens, number of muscles and means for control (Con) and ractopamine treatment (RAC) groups for Warner –Bratzler shear force and cooking loss. **Table S11.** List of ractopamine studies that did not meet the selection criteria, insufficient data or data provided were not appropriate.
(DOCX)

## References

[1] Pringle TD, Calkins CR, Koohmaraie M, Jones SJ (1993) Effects over time of feeding a beta-adrenergic agonist to wether lambs on animal performance, muscle growth, endogenous muscle proteinase activities, and meat tenderness. J Anim Sci 71:636–644. 7681817 10.2527/1993.713636x

[2] Crome PK, McKeith FK, Carr TR, Jones DJ. Mowrey DH, et al (1996) Effect of ractopamine on growth performance, carcass composition, and cutting yields of pigs slaughtered at 107 and 125 kilograms. J Anim Sci 74:709–716. 8727989 10.2527/1996.744709x

[3] Liu CY, Mills SE (1989) Determination of the affinity of ractopamine and clenbuterol for the beta-adrenoceptor of the porcine adipocyte. J Anim Sci 67:2937–2942. 2574169 10.2527/jas1989.67112937x

[4] Dunshea FR (1993) Effect of metabolism modifiers on lipid metabolism in the pig. J Anim Sci 71:1966–1977. 8102358 10.2527/1993.7171966x

[5] Mersmann HJ (1998) Overview of the effects of beta-adrenergic receptor agonists on animal growth including mechanisms of action. J Anim Sci 76:160–172. 9464897 10.2527/1998.761160x

[6] Dunshea FR, D'Souza DN, Pethick DW, Harper GS, Warner RD (2005) Effects of dietary factors and other metabolic modifiers on quality and nutritional value of meat. Meat Sci 71:8–38. 22064049 10.1016/j.meatsci.2005.05.001

[7] Beerman DH (1993) β-Adrenergic agonists and growth. In: Schreibman SMP, Scanes CG, Pang KTP editors. The Endocrinology of growth, development and metabolism in vertebrates. Academic press, San Diego, California. pp. 345–366.

[8] Moody DE, Hancock DL, Anderson DB (2000) Phenethanolamine repartitioning agents. In: D'Mello JPF, editor. Farm animal metabolism and nutrition. CAB Int, New York. pp. 65–95.

[9] Koohmaraie MM, Kent P, Shackelford SD, Veiseth E, Wheeler TL (2002) Meat tenderness and muscle growth: is there any relationship? Meat Sci 62:345–352. 22061610

[10] Cohen J (1969) Statistical power analysis for the behavioral sciences. Academic Press, New York, NY.

[11] DerSimonian R, Laird N (1986) Meta-analysis in clinical trials. Control Clin Trials 7:177–188. 3802833 10.1016/0197-2456(86)90046-2

[12] Lean IJ, Rabiee AR, Duffield TF Dohoo IR (2009) Use of meta-analysis in animal health and reproduction: methods and applications. J Dairy Sci 92:3545–3565. 19620636 10.3168/jds.2009-2140

[13] Egger M, Smith GD (2001) Principles of and procedures for systematic reviews. In: Egger M, Smith GD, ADGeditors. Systematic reviews in health care meta-analysis in context No. 23–42. British Medical Journal Books, London.

[14] Higgins JPT, Thompson SG (2002) Quantifying heterogeneity in a meta-analysis. Stat Med 21:1539–1558. 12111919 10.1002/sim.1186

[15] Knapp G, Hartung J (2003) Improved tests for a random-effects meta-regression with a single covariate. Stat Med 22:2693–2710. 12939780 10.1002/sim.1482

[16] Higgins JPT, Thompson SG (2004) Controlling the risk of spurious findings from meta-regression. Stat Med 23:1663–1682. 15160401 10.1002/sim.1752

[17] Harbord RM, Higgins JPT (2008) Meta-regression in Stata. Stata J 8:493–519.

[18] Harbord RM, Steichen TJ (2004) “METAREG: Stata module to perform meta-analysis regression,” Statistical Software Components S4446201, Boston College Department of Economics, Revised 05 Jan 2009.

[19] Avendano-Reyes L, Torres-Rodriguez V, Meraz-Murillo FJ, Perez-Linares C, Figueroa-Saavedra F, et al (2006) Effects of two beta-adrenergic agonists on finishing performance, carcass characteristics, and meat quality of feedlot steers. J Anim Sci 84:259–3265. 17093218 10.2527/jas.2006-173

[20] Baxa TJ, Hutchenson JP, Miller MF, Brooks JC, Nicholset WT, et al (2010) Additive effects of a steroidal implant and zilpaterol hydrochloride on feedlot performance, carcass characteristics, and skeletal muscle messenger ribonucleic acid abundance in finishing steers. J Anim Sci 88:330–337. 19783693 10.2527/jas.2009-1797

[21] Beckett JL, Delmore RJ, Duff GC, Yates DA, Allen DM, et al (2009) Effects of zilpaterol hydrochloride on growth rates, feed conversion, and carcass traits in calf-fed Holstein steers. J Anim Sci 87:4092–4100. 19717762 10.2527/jas.2009-1808

[22] Elam NA, Vasconcelos JT, Hilton GG, VanOverbeke DL, Lawrence TE, et al (2009) Effect of zilpaterol hydrochloride duration of feeding on performance and carcass characteristics of feedlot cattle. J Anim Sci 87:2133–2141. 19251916 10.2527/jas.2008-1563

[23] Guzman A, Gonzalez-Padilla E, Garces-Yepez P, Rosette-Fernandex JV, Calderon-Robles RC, et al (2012) Reduced response to an estrous induction program in postpartum beef cows treated with zilpaterol and gaining body weight. Ani Reprod Sci 130:1–8. 10.1016/j.anireprosci.2011.12.001 22277845

[24] Holland BP, Krehbiel CR, Hilton GG, Streeter MN, Van Koevering TV, et al (2010) Effect of extended withdrawal of zilpaterol hydrochloride on performance and carcass traits in finishing beef steers. J Anim Sci 88:338–348. 19749012 10.2527/jas.2009-1798

[25] Lawrence TE, Gasch CA, Hutcheson JP Hodgen JM (2011b) Zilpaterol improves feeding performance and fabrication yield of concentrate-finished cull cows. J Anim Sci 89:2170–2175. 21278106 10.2527/jas.2010-3422

[26] Luque LD, Johnson BJ, Martin JN, Miller MF, Hodgen JM, et al (2011) Zilpaterol hydrochloride supplementation has no effect on the shelf life of ground beef. J Anim Sci 89:817–825. 21346139 10.2527/jas.2010-3317

[27] Montgomery JL, Krehbiel CR, Cranston JJ, Yates DA, Hutcheson JP, et al (2009) Dietary zilpaterol hydrochloride. I. Feedlot performance and carcass traits of steers and heifers. J Anim Sci 87:1374–1383. 19098247 10.2527/jas.2008-1162

[28] Montgomery JL, Krehbiel CR, Cranstone JJ, Yates DA, Hutchenson JP, et al (2009) Effects of dietary zilpaterol hydrochloride on feedlot performance and carcass characteristics of beef steers fed with and without monensin and tylosin. J Anim Sci 87:1013–1023. 18997069 10.2527/jas.2008-1169

[29] Neill S, Unruh JA, Marston TT, Jaeger JR, Hunt MC, et al (2009) Effects of implanting and feeding zilpaterol hydrochloride on performance, carcass characteristics, and subprimal beef yields of fed cows. J Anim Sci 89:704–710. 10.2527/jas.2008-1254 18820157

[30] Parr SL, Chung KY, Galyean ML, Hutchenson JP, DiLorenzo N, et al (2011) Performance of finishing beef steers in response to anabolic implant and zilpaterol hydrochloride supplementation. J Anim Sci 89:560–570. 20935134 10.2527/jas.2010-3101

[31] Peterson RK (2011) The effect of crude protein withdrawal and the use of β-agonists on feedlot performance, carcase merit, and theoretical nitrogen retention and excretion for heavy yearling steers, Colorado State University, Fort Collins, Colorado.

[32] Rathmann RJ, Bernhard BC, Swingle RS, Lawrence TE, Nichols WT, et al (2012) Effects of zilpaterol hydrochloride and days on the finishing diet on feedlot performance, carcass characteristics, and tenderness in beef heifers. J Anim Sci 90:3301–3311. 22871933 10.2527/jas.2011-4375

[33] Robles-Estrada JC, Arrison AA, Barreras A, Calderon JF, Figueroa-Saavedra F, et al (2009) Effects of preslaughter withdrawal period on response of feedlot heifers to zilpaterol hydrochloride supplementation: growth performance and carcass characteristics. J Anim Sci 87:1759–1763. 19151149 10.2527/jas.2008-1071

[34] Rodas-Gonzalez A, Pflanzer SB, Garmyn AJ, Martin JN, Brooks JC, et al (2012) Effects of postmortem calcium chloride injection on meat palatability traits of strip loin steaks from cattle supplemented with or without zilpaterolhydrochoride. J Anim Sci 90:3584–3595. 22851240 10.2527/jas.2012-5159

[35] Romero MJM, Pinos-Rodriguez JG, Herrera JC, Garcia AZ, Salem M. **et al.** (2009). Influence of zilpaterol and mineral-yeast mixture on ruminal fermentation and growth performance in finishing steers. J Appl Anim Res 35:77–81.

[36] Scramlin SM, Platter WJ, Gomez RA, Choat WT, McKeith FK, et al (2010) Comparative effects of ractopamine hydrochloride and zilpaterol hydrochloride on growth performance, carcass traits, and longissimus tenderness of finishing steers. J Anim Sci 88:1823–1829. 20042550 10.2527/jas.2009-2405

[37] Strydom PE, Frylinck L, Montgomery JL, Smith MF (2009) The comparison of three beta-agonists for growth performance, carcass characteristics and meat quality of feedlot cattle. Meat Sci 81:557–564. 20416591 10.1016/j.meatsci.2008.10.011

[38] Vasconcelos JT, Rathmann RJ, Reuter RR, Leibovich J, McMeniman JP, et al (2008) Effects of duration of zilpaterol feeding and days on the finishing diet on feedlot cattle performance and carcass traits. J Anim Sci 86:2005–2015. 18469048 10.2527/jas.2008-1032

[39] Garmyn AJ, Shook JN, Van Koevering TV, Beckett JL, Delmore RJ, et al (2010) The effects of zilpaterol hydrochloride on carcass cutability and tenderness of calf-fed Holstein steers. J Anim Sci 88:2476–2485. 20382878 10.2527/jas.2009-2635

[40] Garmyn AJ, Knobel SM, Spivey KS, Hightower LF, Brooks JC, et al (2011) Warner-Bratzler and slice shear force measurements of 3 beef muscles in response to various aging periods after trenbolone acetate and estradiol implants and zilpaterol hydrochloride supplementation of finishing beef steers. J Anim Sci 89:3783–3791. 21680791 10.2527/jas.2011-4134

[41] Hilton GG, Montgomery JL, Krenbiel DA, Yates DA, Hutchenson JP, et al (2009) Effects of feeding zilpaterol hydrochloride with and without monensin and tylosin on carcass cutability and meat palatability of beef steers. J Anim Sci 87:1394–1406. 19028853 10.2527/jas.2008-1170

[42] Kellermeier JD, Tittor AW, Brooks JC, Galyean ML, Yates DA, et al (2009) Effects of zilpaterol hydrochloride with or without an estrogen-trenbolone acetate terminal implant on carcass traits, retail cutout, tenderness, and muscle fiber diameter in finishing steers. J Anim Sci 87:3702–3711. 19502510 10.2527/jas.2009-1823

[43] Lawrence TE, Allen DM, Delmore RJ, Beckett JL, Nichols WT, et al (2011a;Technical note: Feeding zilpaterol hydrochloride to calf-fed Holstein steers improves muscle conformation of top lion steaks. Meat Sci 88:209–211. 21232876 10.1016/j.meatsci.2010.12.031

[44] Brooks JC, Mehaffey JM, Collins JA, Rogers HR, Legako JB, Jet al. (2010) Moisture enhancement and blade tenderization effects on the shear force and palatability of strip loin steaks from beef cattle fed zilpaterol hydrochloride. J Anim Sci 88:1809–1816. 20081069 10.2527/jas.2009-2383

[45] Hansen S, Frylinck L, Strydom PE (2012) The effect of vitamin D 3 supplementation on texture and oxidative stability of beef loins from steers treated with zilpaterol hydrochloride. Meat Sci 90:145–151. 21723673 10.1016/j.meatsci.2011.06.014

[46] Holmer SF, Fernández-Dueñas DM, Scramlin SM, Souza CM, Boler DD, et al (2009b) The effect of zilpaterol hydrochloride on meat quality of calf-fed Holstein steers. J Anim Sci 87:3730–3738. 19648490 10.2527/jas.2009-1838

[47] Leheska JM, Montgomery JL, Krehbiel CR, Yates DA, Hutcheson JP, et al (2009) Dietary zilpaterol hydrochloride. II. Carcass composition and meat palatability of beef cattle. J Anim Sci 87:1384–1393. 18849379 10.2527/jas.2008-1168

[48] Rathmann RJ, Mehaffey JM, Baxa TJ, Nichols WT, Yates DA, et al (2009) Effects of duration of zilpaterol hydrochloride and days on the finishing diet on carcass cutability, composition, tenderness, and skeletal muscle gene expression in feedlot steers. J Anim Sci 87:3686–3701. 19502511 10.2527/jas.2009-1818

[49] Boler DD, Holmer SF, McKeith FK, Killefer J, VanOverbeke DL, et al (2009) Effects of feeding zilpaterol hydrochloride for twenty to forty days on carcass cutability and subprimal yield of calf-fed Holstein steers. J Anim Sci 87:3722–3729. 19574574 10.2527/jas.2009-1830

[50] Brooks JC, Claus HC, Dikeman ME, Shook J, Hilton GG, et al (2009) Effects of zilpaterol hydrochloride feeding duration and postmortem aging on Warner-Bratzlershear force of three muscles from beef steers and heifers. J Anim Sci 87:3764–3769. 19648496 10.2527/jas.2009-1885

[51] Claus HL, Dikeman ME, Murray L, Brooks JC, Shook J, et al (2010) Effects of supplementing feedlot steers and heifers with zilpaterol hydrochloride on Warner-Bratzler shear force interrelationships of steer and heifer longissimus lumborum and heifer triceps brachii and gluteus medius muscles aged for 7, 14 and 21 d. Meat Sci 85:347–355. 20374910 10.1016/j.meatsci.2010.02.002

[52] Cooprider KL, Mitloehner FM, Famula TR, Kebreab E, Zhao, et al. (2011) Feedlot efficiency implications on greenhouse gas emissions and sustainability. J Anim Sci 89:2643–2656. 21398565 10.2527/jas.2010-3539

[53] Delmore RJ, Hodgen JM, Johnson BJ (2010) Perspectives on the application of zilpaterol hydrochloride in the United States beef industry. J Anim Sci 88:2825–2828. 20382871 10.2527/jas.2009-2473

[54] Edrington TS, Callaway TR, Ives SE, Engler MJ, Welsh TH, et al (2006) Effect of ractopamine HCl supplementation on fecal shedding of *Escherichia coli* O157:H7 and Salmonella in feedlot cattle. Current Microbiol 53:340–345. 16972129 10.1007/s00284-006-0200-9

[55] Edrington TS, Farrow RL, Loneragan GH, Ives SE, Engler MJ, et al (2009) Influence of beta-agonists (ractopamine HCl and zilpaterol HCl on fecal shedding of *Escherichia coli* O157:H7 in feedlot cattle. Journal of Food Protect 72:2587–2591. 10.4315/0362-028x-72.12.2587 20003743

[56] Etherton TD (2009) ASAS Centennial Paper: Animal growth and development research: Historical perspectives. J Anim Sci 87:3060–3064. 19465489 10.2527/jas.2009-1805

[57] Gunderson JA, Hunt MC, Houser TA, Boyle EAE, Dikeman ME, et al (2009) Effects of zilpaterol hydrochloride feeding duration on crossbred beef semimembranosus steak color in aerobic or modified atmosphere packaging. J Anim Sci 87:3739–3750. 19465492 10.2527/jas.2009-1843

[58] Gunderson JA, Hunt MC, Houser TA, Boyle EAE, Dikeman ME, et al (2009) Feeding zilpaterol hydrochloride to calf-fed Holsteins has minimal effects on semimembranosus steak color. J Anim Sci 87:3751–3763. 19617511 10.2527/jas.2009-1844

[59] Haneklaus AN, Hodgen JM, Delmore RJ, Lawrence TE, Yates DA, et al (2011) Effects of zilpaterol hydrochloride on retail yields of subprimals from beef and calf-fed Holstein steers. J Anim Sci 89:2867–2877. 21478448 10.2527/jas.2010-3823

[60] Hilton GG, Garmyn AJ, Lawrence TE, Miller MF, Brooks JC, et al (2010) Effect of zilpaterol hydrochloride supplementation on cutability and subprimal yield of beef steer carcasses. J Anim Sci 88:1817–1822. 20190177 10.2527/jas.2009-2386

[61] Holmer SF, Homm JW, Berger LL, Stetzer AJ, Brewer MS, et al (2009b) Realimentation of cull beef cows. II. Meat quality of muscles from the chuck, loin and round in response to diet and enhancement. J. Muscle Foods 20:307–324.

[62] Lawrence TE, Elam NA, Miller MF, Brooks JC, Hilton GG, et al (2010) Predicting red meat yields in carcasses from beef-type and calf-fed Holstein steers using the United States Department of Agriculture calculated yield grade. J Anim Sci 88:2139–2143. 20190173 10.2527/jas.2009-2739

[63] Mehaffey JM, Brooks JC, Rathmann RJ, Alsup EM, Hutcheson JP, et al (2009) Effect of feeding zilpaterol hydrochloride to beef and calf-fed Holstein cattle on consumer palatability ratings. J Anim Sci 87:3712–3721. 19717772 10.2527/jas.2009-1837

[64] Miller EK, Chung KY, Hutcheson JP, Yates DA, Smith SB, et al (2012) Zilpaterol hydrochloride alters abundance of beta-adrenergic receptors in bovine muscle cells but has little effect on de novo fatty acid biosynthesis in bovine subcutaneous adipose tissue explants. J Anim Sci 90:1317–1327. 22079997 10.2527/jas.2011-4589

[65] Rogers HR, Brooks JC, Hunt MC, Hilton GG, VanOverbeke DL, et al (2010) Effects of zilpaterol hydrochloride feeding duration on beef and calf-fed Holstein strip loin steak color. J Anim Sci 88:1168–1183. 19966164 10.2527/jas.2009-2369

[66] Shook JN, VanOverbeke DL, Kinman LA, Krehbiel CR, Holland BP, et al (2009) Effects of zilpaterol hydrochloride and zilpaterol hydrochloride withdrawal time on beef carcass cutability, composition, and tenderness. J Anim Sci 87:3677–3685. 19684279 10.2527/jas.2009-1816

[67] Stephany RW (2001) Hormones in meat: different approaches in the EU and in the USA. Apmis 109:S357–S364. 10.1111/j.1600-0463.2001.tb05787.x 11505585

[68] Strydom PE, Hope-Jones M, Frylinck L, Webb EC (2011) The effects of a beta-agonist treatment, vitamin D3 supplementation and electrical stimulation on meat quality of feedlot steers. Meat Sci 89:462–468. 21658849 10.1016/j.meatsci.2011.05.012

[69] Van Donkersgoed J, Royan G, Berg J, Hutchenson J, Browne M (2011) Comparative effects of zilpaterol hydrochloride and ractopamine hydrochloride on growth performance, carcass characteristics, and longissimus tenderness of feedlot heifers fed barley-based diets. Prof Anim Scient 27:116–121.

[70] Abney CS, Vasconcelos JT, McMeniman JP, Keyser SA, Wilson KR (2007) Effects of ractopamine hydrochloride on performance, rate and variation in feed intake, and acid-base balance in feedlot cattle. J Anim Sci 85:3090–3098. 17609477 10.2527/jas.2007-0263

[71] Allen JD, Ahola JK, Chahine M, Szasz JI, Hunt CW, et al (2009) Effect of preslaughter feeding and ractopamine hydrochloride supplementation on growth performance, carcass characteristics, and end product quality in market dairy cows. J Anim Sci 87:2400–2408. 19359500 10.2527/jas.2008-1630

[72] Boler DD, Shreck AL, Faulkner DB, Killefer J, McKeith FK, et al (2012) Effect of ractopamine hydrochloride (Optaflexx) dose on live animal performance, carcass characteristics and tenderness in early weaned beef steers. Meat Sci 92:458–463. 22824129 10.1016/j.meatsci.2012.05.011

[73] Bryant TC, Engle TE, Galyean ML, Wagner JJ, Tatum JD, et al (2010) Effects of ractopamine and trenbolone acetate implants with or without estradiol on growth performance, carcass characteristics, adipogenic enzyme activity, and blood metabolites in feedlot steers and heifers. J Anim Sci 88:4102–4119. 20729282 10.2527/jas.2010-2901

[74] Glanc DL (2013) Effects of source and level of dietary roughage and ractopamine (Optaflexx) supplementation on growth performance, carcass characteristics and meat quality in beef cattle, The University of Guelph, Guelph, Ontario, Canada.

[75] Gruber SL, Tatum JD, Engle TE, Mitchell MA, Laudert SB, et al (2007) Effects of ractopamine supplementation on growth performance and carcass characteristics of feedlot steers differing in biological type. J Anim Sci 85:1809–1815. 17431043 10.2527/jas.2006-634

[76] Holmer SF, Homm JW, Berger LL, Brewer MS, McKeith FK, et al (2009a) Realimentation of cull beef cows. I. Live performance, carcass traits and muscle characteristics. J Muscle Foods 20:293–306.

[77] Jennings MA (2012) Interaction of Optaflexx and terminal implant window on growth performance and carcass characteristics in heifers fed to harvest, Texas Tech University.

[78] Laudert SB, Vogel GJ, Schroeder AL, Platter WJ, Van Koevering TV (2004) The effect of Optaflexx on Growth Performance and carcass traits of steers, summary of six post-registration studies. In: Health EA, editor. Optaflexx Exchange: Scientific update from Elance Animal Health No. 4. Elanco Animal health, Greenfield, Indiana.

[79] Quinn MJ, Reinhardt CD, Loe ER, Depenbusch BE, Corrigan ME, et al (2008) The effects of ractopamine-hydrogen chloride (Optaflexx) on performance, carcass characteristics, and meat quality of finishing feedlot heifers. J Anim Sci 86:902–908. 18192549 10.2527/jas.2007-0117

[80] Schluter AR (1991) Ractopamine hydrochloride effects on feedlot performance, carcass traits, and chemical composition of feedlot steers, Texas Tech University.

[81] Schroeder AL, Polser DM, Laudert SB, Vogel GJ (2003) The effect of Optaflexx on Growth Performance and carcass traits of steers, five-trial registration summary. In: Health EAeditor. Optaflexx Exchange: Scientific update from Elanco Animal Health No. 1. Elanco Animal health, Greenfield, Indiana.

[82] Schroeder A, Hancock D, Mowrey D, Laudert S, Vogel G, et al (2005) Dose titration of Optaflexx (ractopamine HCl) evaluating the effects on growth performance in feedlot steers. J Anim Sci 83:1.15583036

[83] Sissom EK, Reinhardt CD, Hutcheson JP, Nichols WT, Yates DA, et al (2007) Response to ractopamine-HCl in heifers is altered by implant strategy across days on feed. J Anim Sci 85:2125–2132. 17504961 10.2527/jas.2006-660

[84] Talton CS (2006) Effects of Optaflexx feeding on animal performance, carcass traits, yields of carcass primals and value cuts, and meat tenderness in ovariectomized heifers, University of Georgia, Athens, Georgia.

[85] Vogel GJ, Duff GC, Lehmkuhler J, Beckett JL, Drouillard JS, et al (2009) Effect of ractopamine hydrochloride on growth performance and carcass traits in calf-fed and yearling Holstein steers fed to slaughter. Prof Anim Scient 25:26–32.

[86] Walker DK, Titgemeyer EC, Drouillard JS, Loe ER, Depenbusch BE, et al (2006) Effects of ractopamine and protein source on growth performance and carcass characteristics of feedlot heifers. J Anim Sci 84:2795–2800. 16971581 10.2527/jas.2005-614

[87] Winterholler SJ, Parsons GL, Reinhardt CD, Hutcheson JP, Nichols WT, et al (2007) Response to ractopamine-hydrogen chloride is similar in yearling steers across days on feed. J Anim Sci 85:413–419. 17235026 10.2527/jas.2006-555

[88] Winterholler SJ, Parsons GL, Walker DK, Quinn MJ, Drouillard JS, et al (2008) Effect of feedlot management system on response to ractopamine-HCl in yearling steers. J Anim Sci 86:2401–2414. 18469052 10.2527/jas.2007-0482

[89] Woerner DR, Tatum JD, Engle TE, Belk KE, Couch DW (2011) Effects of sequential implanting and ractopamine hydrochloride supplementation on carcass characteristics and longissimus muscle tenderness of calf-fed steers and heifers. J Anim Sci 89:201–209. 21178182 10.2527/jas.2010-2857

[90] Bass PD, Beckett JL, Delmore RJ Jr (2009) Effects of ractopamine in combination with various hormone implant regimens on growth and carcass attributes in calf-fed Holstein steers. Prof Anim Scient 25:195–201.

[91] Griffin WA, Erickson GE, Dicke BD, Klopfenstein TJ, Cooper RJ, et al (2009a) Effects of ractopamine (Optaflexx) fed in combination with melengestrol acetate on feedlot heifer performance. Prof Anim Scient 25:33–40.

[92] Gruber SL, Tatum JD, Engle TE, Prusa KJ, Laudert SB, et al (2008) Effects of ractopamine supplementation and postmortem aging on longissimus muscle palatability of beef steers differing in biological type. J Anim Sci 86:205–210. 17878276 10.2527/jas.2007-0201

[93] Beerman DH (2002) Beta-Adrenergic receptor agonist modulation of skeletal muscle growth. J Anim Sci 80:E18–E23.

[94] Dijkhuis RD, Johnson DD, Carter JN (2008) Feeding ractopamine hydrochloride to cull cows: effects on carcass composition, Warner-Bratzler shear force, and yield. Prof Anim Scient 24:634–638.

[95] Eisemann JH, Bristol DG (1998) Change in insulin sensitivity or responsiveness is not a major component of the mechanism of action of ractopamine in beef steers. J Nutr 128:505–511. 9482756 10.1093/jn/128.3.505

[96] Gonzalez JM, Carter JN, Johnson DD, Ouellette SE, Johnson SE (2007) Effect of ractopamine-hydrochloride and trenbolone acetate on longissimus muscle fiber area, diameter, and satellite cell numbers in cull beef cows. J Anim Sci 85:1893–1901. 17468415 10.2527/jas.2006-624

[97] Gonzalez JM, Dijkhuis RD, Johnson DD, Carter JN, Johnson SE (2008) Differential response of cull cow muscles to the hypertrophic actions of ractopamine-hydrogen chloride. J Anim Sci 86:3568–3574. 18708600 10.2527/jas.2008-1049

[98] Gonzalez JM, Johnson SE, Thrift TA, Savell JD, Ouellette SE, et al (2009) Effect of ractopamine-hydrochloride on the fiber type distribution and shelf-life of six muscles of steers. J Anim Sci 87:1764–1771. 19181765 10.2527/jas.2008-1469

[99] Griffin WA, Klopfenstein TJ, Erickson GE, Feuz DM, Pol JK, et al (2009b) Effect of sorting and optaflexx supplementation on feedlot performance and profitability of long yearling steers. Prof Anim Scient 25:273–282.

[100] Li C, Jiang J (2012) Preparation of a polyclonal antibody based heterologous indirect competitive ELISA for detecting ractopamine residue. J Food Agric Environ 10:1349–1352.

[101] Paddock ZD, Walker CE, Drouillard JS, Nagaraja TG (2011) Dietary monensin level, supplemental urea, and ractopamine on fecal shedding of *Escherichia coli* O157:H7 in feedlot cattle. J Anim Sci 89:2829–2835. 21512125 10.2527/jas.2010-3793

[102] Shelver WL, Smith DJ (2002) Application of a monoclonal antibody-based enzyme-linked immunosorbent assay for the determination of ractopamine in incurred samples from food animals. J Agr Food Chem 50:2742–2747. 11982392 10.1021/jf011372+

[103] Lean IJ, Celi P, Raadsma H, McNamara J, Rabiee AR (2012) Effects of dietary crude protein on fertility: meta-analysis and meta-regression. Anim Feed Sci Tech 171:31–42.

[104] Rabiee AR, Breinhild K, Scott S, Golder HM, Block E, et al (2012) Effect of fat additions to diets of dairy cattle on milk production and components: a meta-analysis and meta-regression. J Dairy Sci 95:3225–3247. 22612958 10.3168/jds.2011-4895

[105] Tempelman RJ (2009) Assessing experimental designs for research conducted on commercial dairies. J Dairy Sci 92:1–15. 19109258 10.3168/jds.2008-1404

[106] White IR, Thomas J (2005) Standardized mean difference in individually randomized and cluster-randomized trials, with applications to meta-analysis. Clin Trials 2:141–151. 16279136 10.1191/1740774505cn081oa

[107] Zobel G, Schwartzkopf-Genswein KS, Genswein MBA, von Keyselingk MAG (2011) Impact of agonistic interactions on feeding behaviours when beef heifers are fed in a competitive feeding environment. Livest Sci 137:1–9.

[108] Ponnampalam EN, Warner RD, Dunshea FR (2012) Basal and hormone stimulated metabolism in lambs can vary with genotype and diet quality. Domest Anim Endocrin 42:94–102. 10.1016/j.domaniend.2011.10.001 22119112

[109] Loneragan GH, Thomson DU, Scott HM (2014) Increased mortality in groups of cattle administered the β-Adrenergic agonists Ractopamine Hydrochloride and Zilpaterol Hydrochloride. PLoS ONE 9:e91177. 24621596 10.1371/journal.pone.0091177 PMC3951294

