# Supplementary material for: A Meta-Analysis of Zilpaterol and Ractopamine Effects on Feedlot Performance, Carcass Traits and Shear Strength of Meat in Cattle
Source: PLoS One. 2014 Dec 30;9(12):e115904. doi: 10.1371/journal.pone.0115904 (PMC4280124; doi:10.1371/journal.pone.0115904)
Supplement: S1 File — Contains the following files: Table S1. Authors, experimental unit, number of cattle and pens per group, and means for control (Con) and treatment (ZH) groups for final body weight (BW), average daily gain (ADG),dry matter intake (DMI) and Gain/Feed (G/F) ratio. Table S2. Authors, number of cattle per group, experimental unit, number of cattle and carcasses per group for analysing carcass characteristics in experiments with control (Con) and treatment (ZH) groups. Table S3. Authors and study means for control (Con) and treatment (ZH) groups for hot carcass weight (HCW), longissimus muscle area (LMA), ultimate pH and muscle colour score. Table S4. Authors and study means for control (Con) and treatment (ZH) groups for USDA marbling score, fat thickness (mm) and dressing percentage (D%). Table S5. Author, experimental unit, days aged, number of animals, number of pens, number of muscles and study means for control (Con) and treatment (ZL) groups for Warner Bratzler shear force (WBSF)and cooking loss percentage (CL%). Table S6. Zilpaterol studies that did not meet the selection criteria, had insufficient data or data provided were not appropriate. Table S7. Authors, experimental unit, number of cattle and pens per group, and means for control (Con) and ractopamine treatment (RAC) groups for final body weight (BW), average daily gain (ADG), dry matter intake (DMI) and Gain/Feed (G:F) ratio. Table S8. Authors, \and means for control (Con) and ractopamine treatment (RAC) groups for hot carcass weight (HCW), longissimus muscle area (LMA) and ultimate muscle pH. Table S9. Authors and means for control (Con) and ractopaminetreatment (RAC) groups for USDA marbling score, fat thickness (mm) and dressing percentage (D%). Table S10. Authors, experimental unit, days aged, number of animals, number of pens, number of muscles and means for control (Con) and ractopamine treatment (RAC) groups for Warner –Bratzler shear force and cooking loss. Table S11. List of ractopamine studies t [file pone.0115904.s002.docx]

**Table S1.** Authors**,** experimental unit, number of cattle and pens per group, and means for control (Con) and treatment (ZH) groups for final body weight (BW), average daily gain (ADG),dry matter intake (DMI) and Gain/Feed(G/F)ratio

| References | | Authors | Experimental Unit | | |  | No animals/ group | | No animals/pen | No pens/group |  | BW (kg) | | |  |  | ADG (kg/hd/day) | | |  |  | DMI (kg/hd/day) | | |  |  | G/F (DMIDMI) | | |  |
| --- | --- | --- | --- | --- | --- | --- | --- | --- | --- | --- | --- | --- | --- | --- | --- | --- | --- | --- | --- | --- | --- | --- | --- | --- | --- | --- | --- | --- | --- | --- |
|  |  | | | |  | Con | | ZH |  |  | Con | | ZH | SE | | Con | | ZH | SE | | Con | | ZH | SE | | Con | | ZH | SE | |
| 19 | Avendano-Reyes et al., 2006 | | | | Pen | 17 | | 17 | 3 | 6 | 478 | | 498 | 1.7 | | 1.58 | | 2.14 | 0.033 | | 8.51 | | 8.46 | 0.02 | | 0.185 | | 0.253 | 0.004 | |
| 20 | Baxa et al., 2010 | | | | Pen | 570 | | 570 | 90 to 100 | 6 | 599 | | 600 | 9.2 | | 1.81 | | 1.92 | 0.083 | | 10.31 | | 10.21 | 0.27 | | 0.176 | | 0.188 | 0.004 | |
| 20 | Baxa et al., 2010 | | | | Pen | 570 | | 570 | 90 to 100 | 6 | 599 | | 600 | 9.2 | | 1.81 | | 1.92 | 0.083 | | 10.31 | | 10.21 | 0.27 | | 0.176 | | 0.188 | 0.004 | |
| 21 | Beckett et al., 2009-(1) | | | | Pen | 584 | | 587 | 81 to 100 | 6 | 642 | | 646 | 2.9 | | 1.39 | | 1.42 | 0.031 | | 9.87 | | 9.59 | 0.16 | | 0.141 | | 0.148 | 0.004 | |
| 21 | Beckett et al., 2009-(2) | | | | Pen | 584 | | 582 | 81 to 100 | 6 | 642 | | 648 | 2.9 | | 1.39 | | 1.43 | 0.031 | | 9.87 | | 9.60 | 0.16 | | 0.141 | | 0.150 | 0.004 | |
| 21 | Beckett et al., 2009-(3) | | | | Pen | 584 | | 581 | 81 to 100 | 6 | 642 | | 656 | 2.9 | | 1.39 | | 1.50 | 0.031 | | 9.87 | | 9.63 | 0.16 | | 0.141 | | 0.156 | 0.004 | |
| 21 | Beckett et al., 2009-(4) | | | | Pen | 90 | | 90 | 9 | 10 | 588 | | 587 | 0.9 | | 1.33 | | 1.35 | 0.057 | | 9.14 | | 8.85 | 0.16 | | 0.146 | | 0.153 | 0.006 | |
| 21 | Beckett et al., 2009-(5) | | | | Pen | 90 | | 90 | 9 | 10 | 588 | | 594 | 0.9 | | 1.33 | | 1.43 | 0.057 | | 9.14 | | 9.06 | 0.16 | | 0.146 | | 0.158 | 0.006 | |
| 21 | Beckett et al., 2009-(6) | | | | Pen | 90 | | 89 | 9 | 10 | 588 | | 592 | 0.9 | | 1.33 | | 1.41 | 0.057 | | 9.14 | | 8.66 | 0.16 | | 0.146 | | 0.164 | 0.006 | |
| 22 | Elam et al., 2009-(1) | | | | Pen | 1008 | | 1008 | 70 to 98 | 6 to 7 | 568 | | 569 | 12.3 | | 1.03 | | 1.19 | 0.057 | | 9.23 | | 9.17 | 0.12 | | 0.111 | | 0.129 | 0.006 | |
| 22 | Elam et al., 2009-(2) | | | | Pen | 1008 | | 1008 | 71 to 98 | 6 to 7 | 568 | | 566 | 12.3 | | 1.03 | | 1.27 | 0.057 | | 9.23 | | 9.24 | 0.120 | | 0.111 | | 0.138 | 0.006 | |
| 22 | Elam et al., 2009-(3) | | | | Pen | 1008 | | 1008 | 72 to 98 | 6 to 7 | 568 | | 566 | 12.3 | | 1.03 | | 1.29 | 0.057 | | 9.23 | | 9.19 | 0.120 | | 0.111 | | 0.140 | 0.006 | |
| 23 | Guzman et al., 2012 | | | Not Provided | | 39 | | 38 |  |  | 385 | | 395 | 2.2 | | 0.04 | | 0.38 | 0.074 | |  | |  |  | |  | |  |  | |
| 24 | Holland et al., 2010 | | | | Pen | 186 | | 186 | 6 | 32 | 579 | | 584 | 4.0 | | 1.36 | | 1.51 | 0.170 | | 10.07 | | 9.65 | 0.150 | | 0.135 | | 0.157 | 0.016 | |
| 25 | Lawrence et al., 2011b | | | | Pen | 160 | | 160 | 10 | 16 | 619 | | 641 | 10.2 | | 2.17 | | 2.75 | 0.120 | | 17.20 | | 17.3 | 0.300 | | 0.126 | | 0.160 | 0.007 | |
| 26 | Luque et al., 2010 | | | |  | 24 | | 24 |  | 1 |  | |  |  | |  | |  |  | |  | |  |  | |  | |  |  | |
| 27 | Montgomery et al., 2009a-(1) | | | | Pen | 80 | | 80 | 10 | 4 | 564 | | 574 | 19.0 | | 1.08 | | 1.55 | 0.160 | | 8.950 | | 8.78 | 0.410 | | 0.120 | | 0.176 | 0.012 | |
| 27 | Montgomery et al., 2009a-(2) | | | | Pen | 80 | | 80 | 10 | 4 | 566 | | 580 | 19.0 | | 1.20 | | 1.54 | 0.160 | | 9.230 | | 8.99 | 0.410 | | 0.129 | | 0.171 | 0.012 | |
| 27 | Montgomery et al., 2009a-(3) | | | | Pen | 80 | | 80 | 10 | 4 | 510 | | 516 | 24.0 | | 1.15 | | 1.36 | 0.100 | | 8.560 | | 8.07 | 0.580 | | 0.134 | | 0.168 | 0.005 | |
| 27 | Montgomery et al., 2009a-(4) | | | | Pen | 80 | | 80 | 10 | 4 | 513 | | 521 | 24.0 | | 1.12 | | 1.34 | 0.100 | | 8.780 | | 8.34 | 0.580 | | 0.126 | | 0.162 | 0.005 | |
| 28 | Montgomery et al., 2009b | | | | Pen | 940 | | 940 | 94 | 10 | 559 | | 572 | 3.4 | | 1.42 | | 1.62 | 0.054 | | 9.020 | | 8.70 | 0.080 | | 0.156 | | 0.186 | 0.005 | |
| 29 | Neill et al., 2008-(1) | | | | Pen | 12 | | 12 | 6 | 2 | 623 | | 622 | 24.7 | |  | |  |  | |  | |  |  | | 0.130 | | 0.120 | 0.025 | |
| 29 | Neill et al., 2008-(2) | | | | Pen | 12 | | 12 | 6 | 2 | 633 | | 636 | 24.7 | |  | |  |  | |  | |  |  | | 0.140 | | 0.150 | 0.025 | |
| 30 | Parr et al., 2010 | | | | Pen | 27 | | 27 | 4 | 7 | 594 | | 605 | 6.3 | | 1.42 | | 1.49 | 0.033 | | 9.160 | | 9.26 | 0.159 | | 0.155 | | 0.160 | 0.003 | |
| 31 | Peterson, 2011 | | | | Pen | 36 | | 36 | 9 | 4 | 593 | | 599 | 2.7 | | 0.67 | | 0.88 | 0.100 | | 9.16 | | 9.00 | 0.160 | | 0.071 | | 0.096 | 0.010 | |
| 32 | Rathmann et al., 2012 | | | | Pen | 1647 | | 1647 | 77 to 106 | 18 | 543 | | 547 | 3.9 | | 1.47 | | 1.61 | 0.030 | | 9.15 | | 8.97 | 0.040 | | 0.160 | | 0.180 | 0.003 | |
| 33 | Robles-Estrada et al., 2009 | | | | Pen | 32 | | 32 | 8 | 4 | 497 | | 449 | 4.0 | | 1.15 | | 1.83 | 0.110 | | 8.980 | | 9.10 | 0.260 | | 0.129 | | 0.202 | 0.010 | |
| 34 | Rodas-Gonzales et al., 2012 | | | | Pen | 19 | | 20 |  | 12 |  | |  |  | |  | |  |  | |  | |  |  | |  | |  |  | |
| 35 | Romero et al., 2012 | | | | Pen | 5 | | 5 |  | 1 |  | |  |  | | 1.59 | | 1.77 | 0.030 | | 11.10 | | 11.30 | 0.360 | |  | |  |  | |
| 36 | Scramlin et al., 2010 | | | | Pen | 100 | | 100 | 8 | 10 | 547 | | 550 | 1.5 | | 0.95 | | 1.05 | 0.050 | |  | |  |  | | 0.107 | | 0.128 | 0.005 | |
| 37 | Strydom et al., 2009 | | | | Animal | 12 | | 12 |  |  | 569 | | 576 | 6.1 | | 2.10 | | 2.40 | 0.132 | | 13.3 | | 12.5 | 0.289 | |  | |  |  | |
| 38 | Vasconcelos et al., 2008-(1) | | | | Pen | 35 | | 35 | 5 | 7 | 596 | | 607 | 4.9 | | 1.33 | | 1.48 | 0.050 | | 8.900 | | 8.99 | 0.137 | | 0.150 | | 0.165 | 0.004 | |
| 38 | Vasconcelos et al., 2008-(2) | | | | Pen | 35 | | 35 | 5 | 7 | 596 | | 602 | 4.9 | | 1.33 | | 1.49 | 0.050 | | 8.900 | | 8.510 | 0.137 | | 0.150 | | 0.175 | 0.004 | |
| 38 | Vasconcelos et al., 2008-(3) | | | | Pen | 35 | | 35 | 5 | 7 | 596 | | 604 | 4.9 | | 1.33 | | 1.52 | 0.050 | | 8.900 | | 8.500 | 0.137 | | 0.150 | | 0.179 | 0.004 | |

**Table S2.**Authors, number of cattleper group, experimental unit, number of cattle and carcasses per group for analysing carcass characteristics in experiments withcontrol (Con) and treatment (ZH) groups

| Reference | Authors | Experimental unit | No of animals per group  Con ZH | | No of animals per pen | No of pens per group | No of carcass/subprimal/loin per group | No of selected carcasses from each pen |
| --- | --- | --- | --- | --- | --- | --- | --- | --- |
| 19 | Avendano-Reyes et al., 2006 | Animal | 17 | 17 |  | 6 |  |  |
| 20 | Baxa et al., 2010 | Pen | 570 | 570 | 90 to 100 | 6 |  |  |
| 20 | Baxa et al., 2010 | Pen | 570 | 570 | 90 to 100 | 6 |  |  |
| 21 | Beckett et al., 2009-(1) | Pen | 584 | 587 | 81 to 100 | 6 |  |  |
| 21 | Beckett et al., 2009-(2) | Pen | 584 | 582 | 81 to 100 | 6 |  |  |
| 21 | Beckett et al., 2009-(3) | Pen | 584 | 581 | 81 to 100 | 6 |  |  |
| 21 | Beckett et al., 2009-(4) | Pen | 90 | 90 | 9 | 10 |  |  |
| 21 | Beckett et al., 2009-(5) | Pen | 90 | 90 | 9 | 10 |  |  |
| 21 | Beckett et al., 2009-(6) | Pen | 90 | 89 | 9 | 10 |  |  |
| 22 | Elam et al., 2009-(1) | Pen | 1008 | 1008 | 70 to 98 | 6 |  |  |
| 22 | Elam et al., 2009-(2) | Pen | 1008 | 1008 | 71 to 98 | 6 |  |  |
| 22 | Elam et al., 2009-(3) | Pen | 1008 | 1008 | 72 to 98 | 6 |  |  |
| 39 | Garmyn et al., 2010 | Carcass side | 48 | 54 |  |  | 48 |  |
| 40 | Garmyn et al., 2011 | Subprimal | 16 | 16 | 4 | 7 | 16 |  |
| 41 | Hilton et al., 2009 | Pen | 50 | 50 | 5 | 10 |  | 5 |
| 24 | Holland et al., 2010 | Pen | 186 | 186 | 6 | 32 |  | 2 |
| 42 | Kellermeir et al., 2009 | Carcass | 570 | 570 | NA |  | 30 | 7 to 8 |
| 43 | Lawrence et al., 2011a-(1) | Individual loin | 50 | 50 | NA | NA | NA |  |
| 43 | Lawrence et al., 2011a-(2) | Pen | 160 | 160 | 10 | 16 |  |  |
| 26 | Luque et al., 2010 | Carcass | 24 | 24 |  |  | 24 |  |
| 27 | Montgomery et al., 2009a-(1) | Pen | 80 | 80 | 10 | 4 |  | 10 |
| 27 | Montgomery et al., 2009a-(2) | Pen | 80 | 80 | 10 | 4 |  | 10 |
| 27 | Montgomery et al., 2009a-(3) | Pen | 80 | 80 | 10 | 4 |  | 10 |
| 27 | Montgomery et al., 2009a-(4) | Pen | 80 | 80 | 10 | 4 |  | 10 |
| 28 | Montgomery et al., 2009b | Pen | 940 | 940 | 94 | 10 |  | 5 |
| 29 | Neill et al., 2008-(1) | Pen | 12 | 12 | 6 | 2 | NA | NA |
| 29 | Neill et al., 2008-(2) | Pen | 12 | 12 | 6 | 2 | NA | NA |
| 30 | Parr et al., 2010 | Pen | 27 | 27 | 4 | 7 |  | 27 |
| 32 | Rathmann et al., 2012 | Pen | 1647 | 1647 | 77 to 106 | 18 |  |  |
| 33 | Robles-Estrada et al., 2009 | Pen | 32 | 32 | 4 | 4 |  |  |
| 34 | Rodas-Gonzales et al., 2012 | Animal | 19 | 20 | NA | 12 | 12 |  |
| 35 | Romero et al., 2012 | Animal | 5 | 5 | NA | 1 |  |  |
| 36 | Scramlin et al., 2010 | Pen | 100 | 100 | 10 | 10 | 75 | 7 to 8 |
| 37 | Strydom et al., 2009 | Animal | 12 | 12 | 10 |  |  |  |
| 38 | Vasconcelos et al., 2008-(1) | Pen | 35 | 35 | 5 | 7 |  | 5 |
| 38 | Vasconcelos et al., 2008-(2) | Pen | 35 | 35 | 5 | 7 |  | 5 |
| 38 | Vasconcelos et al., 2008-(3) | Pen | 35 | 35 | 5 | 7 |  | 5 |

**Table S3.**Authorsand study means for control (Con) and treatment (ZH) groups for hot carcass weight (HCW), longissimus muscle area (LMA), ultimate pH and muscle colour score

| Reference | Authors |  | HCW (kg) | | |  |  | LMA (cm^2^) | | |  |  | Ultimate pH | | |  |  | Meat colour score | | |  |
| --- | --- | --- | --- | --- | --- | --- | --- | --- | --- | --- | --- | --- | --- | --- | --- | --- | --- | --- | --- | --- | --- |
|  |  | Con | | ZH | SEM | | Con | | ZH | SEM | | Con | | ZH | SEM | | Con | | ZH | SEM | |
| 19 | Avendano-Reyes et al., 2006 | 291.7 | | 313.6 | 1.33 | | 66.8 | | 75.2 | 1.37 | | 5.44 | | 5.43 | 0.007 | |  | |  |  | |
| 20 | Baxa et al., 2010 | 371.1 | | 392.6 | 5.97 | | 90.1 | | 101.1 | 1.28 | |  | |  |  | |  | |  |  | |
| 20 | Baxa et al., 2010 | 382.2 | | 402.6 | 5.97 | | 94.8 | | 107.0 | 1.28 | |  | |  |  | |  | |  |  | |
| 21 | Beckett et al., 2009-(1) | 394.4 | | 406.0 | 2.60 | | 81.2 | | 86.3 | 1.30 | |  | |  |  | | 5.0 | | 5.1 | 0.02 | |
| 21 | Beckett et al., 2009-(2) | 394.4 | | 407.4 | 2.60 | | 81.2 | | 90.1 | 1.30 | |  | |  |  | | 5.0 | | 5.1 | 0.02 | |
| 21 | Beckett et al., 2009-(3) | 394.4 | | 411.6 | 2.60 | | 81.2 | | 89.7 | 1.30 | |  | |  |  | | 5.0 | | 5.1 | 0.02 | |
| 21 | Beckett et al., 2009-(4) | 357.9 | | 367.2 | 6.80 | | 80.0 | | 88.0 | 0.92 | |  | |  |  | | 5.0 | | 5.0 | 0.05 | |
| 21 | Beckett et al., 2009-(5) | 357.9 | | 372.5 | 6.80 | | 80.0 | | 89.4 | 0.92 | |  | |  |  | | 5.0 | | 5.0 | 0.05 | |
| 21 | Beckett et al., 2009-(6) | 357.9 | | 369.0 | 6.80 | | 80.0 | | 89.2 | 0.92 | |  | |  |  | | 5.0 | | 5.0 | 0.05 | |
| 22 | Elam et al., 2009-(1) | 389.3 | | 402.9 | 2.60 | | 87.7 | | 95.6 | 0.68 | |  | |  |  | | 4.9 | | 4.9 | 0.07 | |
| 22 | Elam et al., 2009-(2) | 389.3 | | 405.6 | 2.60 | | 87.7 | | 97.8 | 0.68 | |  | |  |  | | 4.9 | | 4.9 | 0.07 | |
| 22 | Elam et al., 2009-(3) | 389.3 | | 407.5 | 2.60 | | 87.7 | | 98.0 | 0.68 | |  | |  |  | | 4.9 | | 4.9 | 0.07 | |
| 40 | Garmyn et al., 2011 | 373.0 | | 384.0 | 13.50 | | 90.0 | | 94.3 | 2.20 | |  | |  |  | |  | |  |  | |
| 39 | Garmyn et al., 2010 | 351.5 | | 356.0 | 2.17 | | 77.0 | | 79.6 | 1.01 | |  | |  |  | |  | |  |  | |
| 41 | Hilton et al., 2009 |  | |  |  | | 89.0 | | 97.0 | 1.46 | | 5.47 | | 5.48 | 0.020 | | 6.5 | | 6.1 | 0.19 | |
| 24 | Holland et al., 2010 | 369.0 | | 380.0 | 1.50 | | 89.7 | | 94.8 | 0.80 | |  | |  |  | | 4.9 | | 5.0 | 0.09 | |
| 42 | Kellermeir et al., 2009 | 377.0 | | 397.6 | 9.17 | | 87.9 | | 99.0 | 2.66 | |  | |  |  | |  | |  |  | |
| 42 | Kellermeir et al., 2009 | 389.7 | | 400.0 | 9.17 | | 96.4 | | 111.6 | 2.66 | |  | |  |  | |  | |  |  | |
| 43 | Lawrence et al., 2011a-(1) | 393.7 | | 405.2 | 4.27 | | 81.4 | | 86.9 | 1.17 | |  | |  |  | |  | |  |  | |
| 25 | Lawrence et al., 2011b-(2) | 369.2 | | 390.1 | 5.40 | | 86.5 | | 93.3 | 1.10 | |  | |  |  | |  | |  |  | |
| 26 | Luque et al., 2010 |  | |  |  | |  | |  |  | | 5.48 | | 5.53 | 0.420 | |  | |  |  | |
| 27 | Montgomery et al., 2009a-(1) | 353.7 | | 366.7 | 8.30 | | 84.0 | | 91.9 | 1.70 | |  | |  |  | | 5.1 | | 5.2 | 0.14 | |
| 27 | Montgomery et al., 2009a-(2) | 354.8 | | 374.7 | 8.30 | | 85.5 | | 94.1 | 1.70 | |  | |  |  | | 5.1 | | 5.3 | 0.14 | |
| 27 | Montgomery et al., 2009a-(3) | 320.0 | | 331.0 | 14.00 | | 85.0 | | 90.8 | 1.70 | |  | |  |  | | 5.1 | | 5.3 | 0.10 | |

| Reference | Authors |  | HCW (kg) | | |  |  | LMA (cm^2^) | | |  | |  | Ultimate pH | | |  | |  | Meat colour score | | |
| --- | --- | --- | --- | --- | --- | --- | --- | --- | --- | --- | --- | --- | --- | --- | --- | --- | --- | --- | --- | --- | --- | --- |
|  |  | Con | | ZH | SEM | Con | | | ZH | SEM | | Con | | | ZH | SEM | | Con | | | ZH | SEM |
| 27 | Montgomery et al., 2009a-(4) | 323.0 | | 336.0 | 14.00 | 86.8 | | | 93.7 | 1.70 | |  | | |  |  | | 5.1 | | | 5.4 | 0.10 |
| 28 | Montgomery et al., 2009b | 369.0 | | 384.0 | 1.56 | 91.7 | | | 101.1 | 0.85 | |  | | |  |  | | 5.1 | | | 5.1 | 0.05 |
| 29 | Neill et al., 2008-(1) | 364.8 | | 371.7 | 11.61 | 87.8 | | | 87.5 | 3.83 | | 5.60 | | | 5.60 | 0.070 | | 6.1 | | | 6.1 | 0.63 |
| 29 | Neill et al., 2008-(2) | 376.7 | | 380.9 | 11.61 | 92.0 | | | 101.4 | 3.83 | | 5.60 | | | 5.70 | 0.070 | | 5.9 | | | 5.2 | 0.63 |
| 30 | Parr et al., 2010 | 390.0 | | 409.0 | 4.30 |  | | |  |  | |  | | |  |  | |  | | |  |  |
| 31 | Peterson, 2011 | 375.3 | | 384.5 | 4.00 | 83.5 | | | 87.9 | 0.90 | |  | | |  |  | |  | | |  |  |
| 32 | Rathmann et al., 2012 | 344.9 | | 356.0 | 2.67 | 86.2 | | | 91.8 | 0.82 | |  | | |  |  | |  | | |  |  |
| 33 | Robles-Estrada et al., 2009 | 309.0 | | 320.1 | 2.94 | 93.5 | | | 99.4 | 1.83 | |  | | |  |  | |  | | |  |  |
| 34 | Rodas-Gonzales et al., 2012 | 357.8 | | 402.7 | 7.37 | 89.0 | | | 101.1 | 2.54 | |  | | |  |  | |  | | |  |  |
| 36 | Scramlin et al., 2010 | 357.8 | | 370.7 | 1.36 | 34.2 | | | 38.0 | 0.43 | | 5.46 | | | 5.47 | 0.010 | |  | | |  |  |
| 37 | Strydom et al., 2009 | 339.1 | | 353.5 | 4.60 | 75.5 | | | 83.9 | 2.65 | |  | | |  |  | |  | | |  |  |
| 38 | Vasconcelos et al., 2008-(1) | 383.9 | | 401.1 | 3.40 | 90.4 | | | 99.6 | 0.90 | |  | | |  |  | |  | | |  |  |
| 38 | Vasconcelos et al., 2008-(2) | 383.9 | | 400.2 | 3.40 | 90.4 | | | 100.6 | 0.90 | |  | | |  |  | |  | | |  |  |
| 38 | Vasconcelos et al., 2008-(3) | 383.9 | | 402.0 | 3.40 | 90.4 | | | 99.7 | 0.90 | |  | | |  |  | |  | | |  |  |

**Table S4.**Authors and study means for control (Con) and treatment (ZH) groups for USDA marbling score, fat thickness (mm) and dressing percentage (D%)

| Reference | Authors |  | USDA Marbling score | | |  |  | Fat thickness (mm) | | |  |  | D% | | |  |
| --- | --- | --- | --- | --- | --- | --- | --- | --- | --- | --- | --- | --- | --- | --- | --- | --- |
|  |  | Con | | HL | SEM | | Con | | ZH | SEM | | Con | | ZH | SEM | |
| 19 | Avendano-Reyes et al., 2006 |  | |  |  | | 16.5 | | 13.6 | 0.58 | | 61.0 | | 63.0 | 0.22 | |
| 20 | Baxa et al., 2010 | 370 | | 340 | 6.5 | | 15.2 | | 14.0 | 0.33 | | 62.9 | | 65.4 | 0.14 | |
| 20 | Baxa et al., 2010 | 353 | | 324 | 6.5 | | 15.0 | | 13.5 | 0.33 | | 63.3 | | 65.6 | 0.14 | |
| 21 | Beckett et al., 2009-(1) | 475 | | 455 | 5.3 | | 7.6 | | 7.6 | 0.20 | | 61.4 | | 62.9 | 0.16 | |
| 21 | Beckett et al., 2009-(2) | 475 | | 445 | 5.3 | | 7.6 | | 7.1 | 0.20 | | 61.4 | | 62.9 | 0.16 | |
| 21 | Beckett et al., 2009-(3) | 475 | | 452 | 5.3 | | 7.6 | | 7.4 | 3.80 | | 61.4 | | 62.8 | 0.16 | |
| 21 | Beckett et al., 2009-(4) | 423 | | 407 | 8.6 | | 6.9 | | 6.4 | 3.80 | | 60.9 | | 62.5 | 0.24 | |
| 21 | Beckett et al., 2009-(5) | 423 | | 393 | 8.6 | | 6.9 | | 6.1 | 3.80 | | 60.9 | | 62.7 | 0.24 | |
| 21 | Beckett et al., 2009-(6) | 423 | | 391 | 8.6 | | 6.9 | | 5.8 | 0.38 | | 60.9 | | 62.3 | 0.24 | |
| 22 | Elam et al., 2009-(1) | 434 | | 420 | 11.6 | | 15.3 | | 14.3 | 0.46 | | 63.4 | | 64.7 | 0.81 | |
| 22 | Elam et al., 2009-(2) | 434 | | 406 | 11.61 | | 15.3 | | 14.0 | 0.46 | | 63.4 | | 65.0 | 0.81 | |
| 22 | Elam et al., 2009-(3) | 434 | | 402 | 11.61 | | 15.3 | | 13.9 | 0.46 | | 63.4 | | 65.2 | 0.81 | |
| 39 | Garmyn et al., 2010 | 426 | | 440 | 10.10 | | 8.9 | | 8.7 | 3.63 | |  | |  |  | |
| 40 | Garmyn et al., 2011 | 492 | | 524 | 26.1 | | 16.4 | | 14.1 | 1.20 | |  | |  |  | |
| 41 | Hilton et al., 2009 | 435 | | 376 | 12.5 | | 11.8 | | 9.1 | 0.20 | |  | |  |  | |
| 24 | Holland et al., 2010 | 440 | | 424 | 13.5 | | 13.6 | | 13.1 | 0.40 | |  | |  |  | |
| 42 | Kellermeir et al., 2009 | 41.5 | | 37.160 | 2.2 | | 16.4 | | 14.3 | 0.65 | |  | |  |  | |
| 42 | Kellermeir et al., 2009 | 35.6 | | 37.2 | 2.2 | | 13.5 | | 12.8 | 0.65 | |  | |  |  | |
| 43 | Lawrence et al., 2011a-(1) |  | |  |  | | 8.7 | | 8.0 | 0.50 | |  | |  |  | |
| 25 | Lawrence et al., 2011b-(2) | 39 | | 36 | 0.05 | | 13.9 | | 14.1 | 0.70 | | 59.5 | | 61.0 | 0.22 | |
| 26 | Luque et al., 2010 |  | |  |  | |  | |  |  | |  | |  |  | |
| 27 | Montgomery et al., 2009a-(1) | 461 | | 432 | 17.0 | | 13.1 | | 12.0 | 1.10 | | 62.7 | | 64.0 | 1.20 | |
| 27 | Montgomery et al., 2009a-(2) | 462 | | 398 | 17.0 | | 12.6 | | 12.0 | 1.10 | | 62.8 | | 64.6 | 1.20 | |
| 27 | Montgomery et al., 2009a-(3) | 461 | | 457 | 23.0 | | 13.9 | | 13.0 | 0.90 | | 62.7 | | 64.2 | 1.10 | |
| 27 | Montgomery et al., 2009a-(4) | 470 | | 437 | 23.0 | | 13.5 | | 13.5 | 0.90 | | 63.0 | | 64.6 | 1.10 | |
| 28 | Montgomery et al., 2009b | 422 | | 391 | 4.9 | | 11.8 | | 10.5 | 0.31 | | 64.8 | | 66.0 | 0.11 | |
| 29 | Neill et al., 2008-(1) | 426 | | 459 | 39.2 | | 10.7 | | 9.5 | 0.15 | | 59.8 | | 58.500 | 0.71 | |
| 29 | Neill et al., 2008-(2) | 435 | | 414.0 | 39.2 | | 9.2 | | 10.3 | 0.15 | | 59.6 | | 60.100 | 0.71 | |
| 30 | Parr et al., 2010 | 487 | | 507.0 | 14.9 | | 15.5 | | 15.7 | 0.07 | |  | |  |  | |
| 31 | Peterson, 2011 | 432 | | 409 | 5.5 | | 12.7 | | 11.9 | 0.30 | | 63.4 | | 64.4 | 0.44 | |
| 32 | Rathmann et al., 2012 | 442 | | 437 | 2.4 | | 15.4 | | 14.6 | 0.04 | | 63.5 | | 65.1 | 0.10 | |
| 33 | Robles-Estrada et al., 2009 | 3.560 | | 3.280 | 0.270 | | 8.5 | | 6.4 | 0.08 | | 62.2 | | 64.2 | 0.45 | |
| 34 | Rodas-Gonzales et al., 2012 | 356 | | 362 | 9.4 | | 12.0 | | 12.1 | 0.12 | |  | |  |  | |
| 35 | Romero et al., 2012 |  | |  |  | |  | |  |  | | 54.4 | | 58.6 | 0.44 | |
| 36 | Scramlin et al., 2010 | 546 | | 525 | 8.7 | | 12.4 | | 10.7 | 0.05 | | 65.5 | | 67.4 | 0.18 | |
| 37 | Strydom et al., 2009 | 221 | | 197 | 9.9 | | 14.2 | | 9.1 | 1.42 | |  | |  |  | |
| 38 | Vasconcelos et al., 2008-(1) | 451 | | 420 | 12.6 | | 14.9 | | 13.5 | 0.05 | | 64.4 | | 66.1 | 0.23 | |
| 38 | Vasconcelos et al., 2008-(2) | 451 | | 405 | 12.6 | | 14.9 | | 12.4 | 0.05 | | 64.4 | | 66.4 | 0.23 | |
| 38 | Vasconcelos et al., 2008-(3) | 451 | | 397 | 12.6 | | 14.9 | | 12.2 | 0.05 | | 64.4 | | 66.6 | 0.23 | |

Marbling score units that differed were excluded from the weighted mean difference estimates

**Table S5.**Author, experimental unit, days aged, number of animals, number of pens, number of muscles and study means for control (Con) and treatment (ZL) groups for Warner Bratzler shear force (WBSF)and cooking loss percentage (CL%)

| Refer | Authors | Experimental | | | Days | No of animals | | |  | No of pens | | |  | No of muscles | |  |  | WBSF (kg) | | |  |  | CL% | | |  |
| --- | --- | --- | --- | --- | --- | --- | --- | --- | --- | --- | --- | --- | --- | --- | --- | --- | --- | --- | --- | --- | --- | --- | --- | --- | --- | --- |
| Ence |  | | | Unit | aged | Con | | ZH | | Con | | ZH | | Con | ZH | | Con | | ZH | SE | | Con | | ZH | SE | |
| 19 | Avendano-Reyes et al., 2006 | | | Animal | 5 & 14 | | 17 | 17 | | 6 | 6 | | | 17 | 17 | | 4.40 | | 5.11 | 0.136 | |  | |  |  | |
| 44 | Brooks et al., 2010- (1) | | | Pen | 7 | |  |  | |  |  | | | 30 | 30 | | 3.08 | | 3.58 | 0.130 | |  | |  |  | |
| 44 | Brooks et al., 2010- (2) | | | Pen | 7 | |  |  | |  |  | | | 30 | 30 | | 3.08 | | 3.77 | 0.130 | |  | |  |  | |
| 44 | Brooks et al., 2010- (3) | | | Pen | 7 | |  |  | |  |  | | | 30 | 30 | | 3.08 | | 3.63 | 0.130 | |  | |  |  | |
| 44 | Brooks et al., 2010- (4) | | | Pen | 14 | |  |  | |  |  | | | 30 | 30 | | 2.88 | | 3.42 | 0.130 | |  | |  |  | |
| 44 | Brooks et al., 2010- (5) | | | Pen | 14 | |  |  | |  |  | | | 30 | 30 | | 2.88 | | 3.50 | 0.130 | |  | |  |  | |
| 44 | Brooks et al., 2010- (6) | | | Pen | 14 | |  |  | |  |  | | | 30 | 30 | | 2.88 | | 3.30 | 0.130 | |  | |  |  | |
| 44 | Brooks et al., 2010- (7) | | | Pen | 21 | |  |  | |  |  | | | 30 | 30 | | 2.84 | | 2.99 | 0.130 | |  | |  |  | |
| 44 | Brooks et al., 2010- (8) | | | Pen | 21 | |  |  | |  |  | | | 30 | 30 | | 2.84 | | 3.23 | 0.130 | |  | |  |  | |
| 44 | Brooks et al., 2010- (9) | | | Pen | 21 | |  |  | |  |  | | | 30 | 30 | | 2.84 | | 3.01 | 0.130 | |  | |  |  | |
| 39 | Garmyn et al., 2010- (6) | | Carcass side | |  | | 48 | 54 | |  |  | | | 48 | 54 | | 3.28 | | 4.10 | 0.111 | | 22.5 | | 22.8 | 0.30 | |
| 40 | Garmyn et al., 2011- (1) | | | Subprimal | 7 | |  |  | | 7 | 7 | | | 77 | 77 | | 2.43 | | 3.58 | 0.260 | | 10.6 | | 12.5 | 0.40 | |
| 40 | Garmyn et al., 2011- (2) | | | Subprimal | 14 | |  |  | | 7 | 7 | | | 77 | 77 | | 2.55 | | 3.59 | 0.220 | | 13.6 | | 14.8 | 0.50 | |
| 40 | Garmyn et al., 2011- (3) | | | Subprimal | 21 | |  |  | | 7 | 7 | | | 77 | 77 | | 2.50 | | 3.29 | 0.200 | | 15.1 | | 17.3 | 1.10 | |
| 40 | Garmyn et al., 2011- (4) | | | Subprimal | 28 | |  |  | | 7 | 7 | | | 77 | 77 | | 1.87 | | 2.58 | 0.290 | | 10.4 | | 12.4 | 0.90 | |
| 40 | Garmyn et al., 2011- (5) | | | Subprimal | 35 | |  |  | | 7 | 7 | | | 77 | 77 | | 2.60 | | 2.89 | 0.170 | | 11.6 | | 12.1 | 1.20 | |
| 46 | Hansen et al., 2011- (1) | | | Animal | 7 | | 10 | 10 | |  |  | | | 10 | 10 | | 3.50 | | 4.30 | 0.242 | |  | |  |  | |
| 46 | Hansen et al., 2011- (2) | | | Animal | 14 | | 10 | 10 | |  |  | | | 10 | 10 | | 2.40 | | 3.70 | 0.242 | |  | |  |  | |
| 46 | Hansen et al., 2012- (1) | | | Animal | 7 | | 10 | 10 | | 1 | 1 | | | 10 | 10 | | 3.50 | | 4.30 | 0.242 | |  | |  |  | |
| 46 | Hansen et al., 2012- (2) | | | Animal | 14 | | 10 | 10 | | 1 | 1 | | | 10 | 10 | | 2.40 | | 3.70 | 0.242 | |  | |  |  | |
| 41 | Hilton et al., 2009- (1) | | | Pen | 7 | | 50 | 50 | | 10 | 10 | | | 50 | 50 | | 3.63 | | 4.94 | 0.170 | | 15.5 | | 16.5 | 0.62 | |
| 41 | Hilton et al., 2009- (2) | | | Pen | 14 | | 50 | 50 | | 10 | 10 | | | 50 | 50 | | 3.56 | | 4.00 | 0.130 | |  | |  |  | |
| 41 | Hilton et al., 2009- (3) | | | Pen | 21 | | 50 | 50 | | 10 | 10 | | | 50 | 50 | | 3.20 | | 3.47 | 0.110 | |  | |  |  | |
| 46 | Holmer et al., 2009a- (1) | | | Carcass |  | | 90 | 90 | | 10 | 10 | | | 90 | 90 | | 2.80 | | 3.60 | 0.170 | |  | |  |  | |
| 46 | Holmer et al., 2009a- (2) | | | Carcass |  | | 90 | 90 | | 10 | 10 | | | 90 | 90 | | 2.80 | | 4.00 | 0.170 | |  | |  |  | |
| 42 | Kellermeir et al., 2009- (1) | | | Carcass | 7 | | 570 | 570 | | 4 | 4 | | | 30 | 30 | | 3.38 | | 5.05 | 0.201 | | 22.3 | | 21.2 | 0.57 | |
| 42 | Kellermeir et al., 2009- (2) | | | Carcass | 14 | |  |  | |  |  | | | 30 | 30 | | 2.83 | | 4.12 | 0.156 | |  | |  |  | |
| 42 | Kellermeir et al., 2009- (3) | | | Carcass | 21 | |  |  | |  |  | | | 30 | 30 | | 2.68 | | 4.08 | 0.225 | |  | |  |  | |
| 47 | Leheska et al., 2009- (1) | | | Pen | 28 | | 120 | 120 | | 12 | 12 | | | 24 | 24 | | 3.19 | | 3.91 | 0.260 | | 13.7 | | 14.2 | 0.46 | |
| 47 | Leheska et al., 2009- (2) | | | Pen | 28 | | 120 | 120 | | 12 | 12 | | | 24 | 24 | | 3.26 | | 3.98 | 0.260 | | 14.4 | | 14.3 | 0.46 | |
| 47 | Leheska et al., 2009- (3) | | | Pen | 28 | | 120 | 120 | | 12 | 12 | | | 24 | 24 | | 3.53 | | 4.37 | 0.370 | | 13.4 | | 14.5 | 0.43 | |
| 47 | Leheska et al., 2009- (4) | | | Pen | 28 | | 120 | 120 | | 12 | 12 | | | 24 | 24 | | 3.28 | | 4.05 | 0.370 | | 14.5 | | 14.0 | 0.43 | |
| 48 | Rathmann et al., 2009- (1) | | | Pen | 7 | | 7 | 7 | | 7 | 7 | | | 7 | 7 | | 3.54 | | 4.94 | 0.230 | |  | |  |  | |
| 48 | Rathmann et al., 2009- (3) | | | Pen | 7 | | 7 | 7 | | 7 | 7 | | | 7 | 7 | | 3.54 | | 5.85 | 0.230 | |  | |  |  | |
| 48 | Rathmann et al., 2009- (4) | | | Pen | 14 | | 7 | 7 | | 7 | 7 | | | 7 | 7 | | 3.37 | | 4.73 | 0.230 | |  | |  |  | |
| 48 | Rathmann et al., 2009- (5) | | | Pen | 14 | | 7 | 7 | | 7 | 7 | | | 7 | 7 | | 3.37 | | 5.22 | 0.230 | |  | |  |  | |
| 48 | Rathmann et al., 2009- (6) | | | Pen | 14 | | 7 | 7 | | 7 | 7 | | | 7 | 7 | | 3.37 | | 4.85 | 0.230 | |  | |  |  | |
| 48 | Rathmann et al., 2009- (7) | | | Pen | 21 | | 7 | 7 | | 7 | 7 | | | 7 | 7 | | 3.12 | | 4.05 | 0.230 | |  | |  |  | |
| 48 | Rathmann et al., 2009- (8) | | | Pen | 21 | | 7 | 7 | | 7 | 7 | | | 7 | 7 | | 3.12 | | 4.65 | 0.230 | |  | |  |  | |
| 48 | Rathmann et al., 2009- (9) | | | Pen | 21 | | 7 | 7 | | 7 | 7 | | | 7 | 7 | | 3.12 | | 4.70 | 0.230 | |  | |  |  | |
| 48 | Rathmann et al., 2009-(2) | | | Pen | 7 | | 7 | 7 | | 7 | 7 | | | 7 | 7 | | 3.54 | | 5.71 | 0.230 | |  | |  |  | |
| 32 | Rathmann et al., 2012- (1) | | | Animal | 7 | | 60 | 60 | | 2 | 2 | | | 60 | 60 | | 3.47 | | 4.25 | 0.070 | |  | |  |  | |
| 32 | Rathmann et al., 2012- (2) | | | Animal | 7 | | 60 | 60 | | 2 | 2 | | | 60 | 60 | | 3.05 | | 3.57 | 0.050 | |  | |  |  | |
| 32 | Rathmann et al., 2012- (3) | | | Animal | 7 | | 60 | 60 | | 2 | 2 | | | 60 | 60 | | 3.03 | | 3.50 | 0.050 | |  | |  |  | |
| 34 | Rodas-Gonzales et al., 2012 (1) | | | Animal | 7 | | 19 | 20 | | 12 | 12 | | | 19 | 20 | | 3.65 | | 4.45 | 0.200 | |  | |  |  | |
| 34 | Rodas-Gonzales et al., 2012 (2) | | | Animal | 14 | | 19 | 20 | | 12 | 12 | | | 19 | 20 | | 3.53 | | 4.08 | 0.140 | |  | |  |  | |
| 34 | Rodas-Gonzales et al., 2012 (3) | | | Animal | 21 | | 19 | 20 | | 12 | 12 | | | 19 | 20 | | 3.29 | | 3.71 | 0.150 | |  | |  |  | |
| 34 | Rodas-Gonzales et al., 2012 (4) | | | Animal | 28 | | 19 | 20 | | 12 | 12 | | | 19 | 20 | | 2.95 | | 3.50 | 0.110 | |  | |  |  | |
| 37 | Strydom et al., 2009 | | | Animal | 2 | | NA | NA | |  |  | | |  |  | | 4.60 | | 5.40 | 0.109 | |  | |  |  | |

**Table S6.**Zilpaterol studies that did not meet the selection criteria, had insufficient data or data provided were not appropriate

| Reference | Studies | Reasons for exclusion |
| --- | --- | --- |
| 49 | Boler et al., 2009 | Did not meet the selection criteria and data were not appropriate |
| 50 | Brooks et al., 2009 | Did not meet the selection criteria and data were not appropriate |
| 51 | Claus et al., 2010 | Insufficient data (data presented in figures not in the tables or text) |
| 52 | Cooprider et al., 2011 | Review – not relevant |
| 53 | Delmore et al., 2010 | Review article |
| 06 | Dunshea et al., 2005 | Review article |
| 54 | Edrington et al., 2006 | Did not meet the selection criteria and data were not appropriate |
| 55 | Edrington et al., 2009 | Did not meet the selection criteria and data were not appropriate |
| 56 | Etherton 2009 | Review article |
| 57 | Gunderson et al., 2009 a | Did not meet the selection criteria and data were not appropriate |
| 58 | Gunderson et al., 2009 b | Did not meet the selection criteria and data were not appropriate |
| 59 | Haneklaus et al., 2011 | Did not meet the selection criteria and data were not appropriate |
| 60 | Hilmer et al., 2010 | Data were not appropriate |
| 61 | Holmer et al., 2009b | Data were not appropriate |
| 62 | Lawrence et al., 2010 | Did not meet the selection criteria and data were not appropriate |
| 63 | Mehaffey et al., 2009 | Did not meet the selection criteria and data were not appropriate |
| 5 | Mersmann 1998 | Review article |
| 64 | Miller et al., 2012 | Did not meet the selection criteria and data were not appropriate |
| 65 | Rogers et al., 2010 | Did not meet the selection criteria and data were not appropriate |
| 66 | Shook et al., 2009 | Data were not appropriate (data of Holland et al., 2010 were re-analysed for this study) |
| 67 | Stephany 2001 | Review article (book chapter) |
| 68 | Strydom et al., 2011 | Did not meet the selection criteria and data were not appropriate |
| 69 | Van Donkersgoed et al., 2011 | There was no negative control (zilpaterol*vs*. ractopamine) |

**Table S7.**Authors**,** experimental unit, number of cattle and pens per group, and means for control (Con) and ractopamine treatment (RAC) groups for final body weight (BW), average daily gain (ADG), dry matter intake (DMI) and Gain/Feed (G:F) ratio

| Reference | | Author | | Experimental | No of animals /group | | | |  | No of animal/ pen | No of pens/ group |  | BW  (kg) | | |  |  | ADG (kg/hd/day) | | |  |  | DMI  (kg/hd/day) | | |  |  | G:F  ratio | | |  |
| --- | --- | --- | --- | --- | --- | --- | --- | --- | --- | --- | --- | --- | --- | --- | --- | --- | --- | --- | --- | --- | --- | --- | --- | --- | --- | --- | --- | --- | --- | --- | --- |
|  |  | | | Unit | Con | | | RAC | |  |  | Con | | RAC | SE | | Con | | RAC | SE | | Con | | RAC | SE | | Con | | RAC | SE | |
| 70 | Abney et al., 2007 (1) | | | Pen | 1 | | | 40 | | 40 | 5 | 585 | | 585 | 11.8 | | 1.04 | | 1.22 | 0.090 | | 8.83 | | 8.80 | 0.280 | | 0.120 | | 0.140 | 0.007 | |
| 70 | Abney et al., 2007 (2) | | | Pen | 1 | | | 40 | | 40 | 5 | 585 | | 592 | 11.8 | | 1.04 | | 1.19 | 0.090 | | 8.83 | | 9.02 | 0.280 | | 0.120 | | 0.130 | 0.007 | |
| 70 | Abney et al., 2007 (3) | | | Pen | 1 | | | 40 | | 40 | 5 | 592 | | 592 | 11.8 | | 1.21 | | 1.30 | 0.090 | | 9.16 | | 9.37 | 0.280 | | 0.130 | | 0.140 | 0.007 | |
| 70 | Abney et al., 2007 (4) | | | Pen | 1 | | | 40 | | 40 | 5 | 592 | | 601 | 11.8 | | 1.21 | | 1.53 | 0.090 | | 9.16 | | 9.46 | 0.280 | | 0.130 | | 0.160 | 0.007 | |
| 70 | Abney et al., 2007 (5) | | | Pen | 1 | | | 40 | | 40 | 5 | 580 | | 599 | 11.8 | | 1.12 | | 1.47 | 0.090 | | 9.06 | | 9.58 | 0.280 | | 0.120 | | 0.150 | 0.007 | |
| 70 | Abney et al., 2007 (6) | | | Pen | 1 | | | 40 | | 40 | 5 | 580 | | 593 | 11.8 | | 1.12 | | 1.39 | 0.090 | | 9.06 | | 9.22 | 0.280 | | 0.120 | | 0.150 | 0.007 | |
| 71 | Allen et al., 2009 | | | Single pens | | | 0 | 8 | | 9 | 1 | 760 | | 760 | 26.0 | | 1.08 | | 1.00 | 0.195 | | 14.50 | | 14.80 | 0.600 | | 0.076 | | 0.067 | 0.011 | |
| 19 | Avendano-Reyes et al., 2006 | | | Pen | 1 | | | 17 | | 18 | 3 | 478 | | 489 | 1.7 | | 1.58 | | 2.08 | 0.033 | | 8.51 | | 8.37 | 0.024 | | 0.185 | | 0.248 | 0.004 | |
| 72 | Boler et al., 2012 (1) | | | Pen | 0 | | | 47 | | 48 | 7 to 8 | 521 | | 536 | 1.9 | | 0.96 | | 1.49 | 0.070 | | 10.09 | | 10.08 | 0.120 | |  | |  |  | |
| 72 | Boler et al., 2012 (2) | | | Pen | 0 | | | 47 | | 48 | 7 to 8 | 521 | | 535 | 1.9 | | 0.96 | | 1.48 | 0.070 | | 10.09 | | 9.76 | 0.120 | |  | |  |  | |
| 73 | Bryant et al., 2010 (1) | | | Pen | 1 | | | 54 | | 54 | 9 | 574 | | 577 | 10.1 | | 0.99 | | 1.25 | 0.091 | | 9.37 | | 9.32 | 0.225 | | 0.105 | | 0.133 | 0.008 | |
| 73 | Bryant et al., 2010 (2) | | | Pen | 1 | | | 54 | | 54 | 9 | 574 | | 580 | 10.1 | | 0.99 | | 1.24 | 0.091 | | 9.37 | | 9.20 | 0.225 | | 0.105 | | 0.134 | 0.008 | |
| 73 | Bryant et al., 2010 (3) | | | Pen | 0 | | | 24 | | 24 | 1 | 501 | | 511 | 10.2 | | 0.61 | | 0.98 | 0.086 | | 7.71 | | 7.78 | 0.162 | | 0.078 | | 0.125 | 0.011 | |
| 74 | Glanc, 2013 (1) | | | Pen | 0 | | | 11 | | 11 | 9 |  | |  |  | | 1.78 | | 2.04 | 0.070 | |  | |  |  | |  | |  |  | |
| 74 | Glanc, 2013 (2) | | | Pen | 0 | | | 11 | | 11 | 9 |  | |  |  | | 1.71 | | 1.93 | 0.070 | |  | |  |  | |  | |  |  | |
| 74 | Glanc, 2013 (3) | | | Pen | 0 | | | 11 | | 11 | 9 |  | |  |  | | 1.81 | | 1.68 | 0.070 | |  | |  |  | |  | |  |  | |
| 74 | Glanc, 2013 (4) | | | Pen | 0 | | | 11 | | 11 | 9 |  | |  |  | | 1.77 | | 1.83 | 0.070 | |  | |  |  | |  | |  |  | |
| 74 | Glanc, 2013 (5) | | | Pen | 0 | | | 11 | | 11 | 9 |  | |  |  | | 1.80 | | 1.72 | 0.070 | |  | |  |  | |  | |  |  | |
| 74 | Glanc, 2013 (6) | | | Pen | 0 | | | 11 | | 11 | 9 |  | |  |  | | 1.65 | | 1.80 | 0.070 | |  | |  |  | |  | |  |  | |
| 75 | Gruber et al., 2007 | | | Pen | 1 | | | 208 | | 209 | 10 | 561 | | 569 | 9.1 | | 1.50 | | 1.73 | 0.090 | | 10.30 | | 10.20 | 0.280 | | 0.145 | | 0.170 | 0.005 | |
| 76 | Holmer et al., 2009c (1) | | | Pen | 1 | | | 19 | | 19 | 5 | 516. | | 651 | 21.3 | | 0.61 | | 2.00 | 0.207 | |  | |  |  | |  | |  |  | |
| 76 | Holmer et al., 2009c(2) | | | Pen | 1 | | | 19 | | 19 | 5 | 661 | | 651 | 21.3 | | 1.90 | | 2.00 | 0.207 | | 13.89 | | 15.32 | 0.868 | | 0.144 | | 0.135 | 0.015 | |
| 77 | Jennings, 2011 | | | Pen | 1 | | | 27 | | 27 | 3 | 548 | | 547 | 3.7 | | 1.32 | | 1.46 | 0.050 | | 8.70 | | 8.75 | 0.120 | | 0.150 | | 0.170 | 0.010 | |
| 78 | Laudert et al., 2004 (1) | | | Pen | 1 | | | 2413 | | 2132 | 76 | 587 | | 590 | 3.1 | | 1.38 | | 1.51 | 0.110 | | 9.08 | | 9.03 | 0.200 | | 0.152 | | 0.167 | 0.009 | |
| 78 | Laudert et al., 2004 (2) | | | Pen | 1 | | | 2413 | | 2419 | 55 | 587 | | 594 | 3.1 | | 1.38 | | 1.51 | 0.110 | | 9.08 | | 9.04 | 0.200 | | 0.152 | | 0.179 | 0.009 | |
| 31 | Peterson, 2011 | | | Pen | 1 | | | 36 | | 36 | 9 | 593 | | 599 | 2.7 | | 0.67 | | 0.91 | 0.100 | | 9.16 | | 9.26 | 0.160 | |  | |  |  | |
| 79 | Quinn et al., 2008 (1) | | | Pen | 1 | | | 150 | | 152 | 12 to 13 | 527 | | 529 | 2.7 | | 1.69 | | 1.80 | 0.050 | | 10.10 | | 9.86 | 0.110 | | 0.167 | | 0.183 | 0.005 | |
| 79 | Quinn et al., 2008 (2) | | | Pen | 1 | | | 57 | | 56 | 5 to 6 |  | |  |  | |  | |  |  | | 8.20 | | 8.20 | 0.120 | |  | |  |  | |
| 79 | Quinn et al., 2008 (3) | | | Pen | 1 | | | 57 | | 57 | 5 to 6 |  | |  |  | |  | |  |  | | 8.20 | | 7.70 | 0.120 | |  | |  |  | |
| 79 | Quinn et al., 2008 (4) | | | Pen | 1 | | | 57 | | 55 | 5 to 6 |  | |  |  | |  | |  |  | | 8.20 | | 8.20 | 0.120 | |  | |  |  | |
| 80 | Schluter (1991) (1) | | | Pen | 1 | | | 40 | | 40 | 8 | 492 | | 497 | 2.6 | | 0.92 | | 0.95 | 0.040 | |  | |  |  | |  | |  |  | |
| 80 | Schluter (1991) (2) | | | Pen | 1 | | | 40 | | 40 | 8 | 492 | | 503 | 2.6 | | 0.92 | | 1.15 | 0.040 | |  | |  |  | |  | |  |  | |
| 80 | Schluter (1991) (3) | | | Pen | 1 | | | 40 | | 40 | 8 | 492 | | 504 | 2.6 | | 0.92 | | 1.17 | 0.040 | |  | |  |  | |  | |  |  | |
| 81 | Schroeder et al., 2003 (1) | | | Pen | 1 | | | 215 | | 219 | 8 to 10 | 576 | | 583 | 20.9 | | 1.27 | | 1.49 | 0.150 | | 9.87 | | 9.99 | 0.490 | | 0.126 | | 0.147 | 0.009 | |
| 81 | Schroeder et al., 2003 (2) | | | Pen | 1 | | | 215 | | 219 | 8 to 10 | 576 | | 583 | 20.9 | | 1.27 | | 1.52 | 0.150 | | 9.87 | | 9.87 | 0.490 | | 0.126 | | 0.152 | 0.009 | |
| 81 | Schroeder et al., 2003 (3) | | | Pen | 1 | | | 215 | | 219 | 8 to 10 | 576 | | 586 | 20.9 | | 1.27 | | 1.60 | 0.150 | | 9.87 | | 9.91 | 0.490 | | 0.126 | | 0.159 | 0.009 | |
| 82 | Schroeder et al., 2005 (1) | | | Pen | 1 | | | 215 | | 215 | 7 to 10 | 528 | | 531 | 15.8 | | 1.24 | | 1.34 | 0.120 | | 9.38 | | 9.41 | 0.370 | | 0.133 | | 0.142 | 0.008 | |
| 82 | Schroeder et al., 2005 (2) | | | Pen | 1 | | | 215 | | 215 | 7 to 10 | 528. | | 535 | 15.8 | | 1.24 | | 1.46 | 0.120 | | 9.38 | | 9.53 | 0.370 | | 0.133 | | 0.153 | 0.008 | |
| 82 | Schroeder et al., 2005 (3) | | | Pen | 1 | | | 215 | | 215 | 7 to 10 | 528 | | 538 | 15.8 | | 1.24 | | 1.50 | 0.120 | | 9.38 | | 9.39 | 0.370 | | 0.133 | | 0.159 | 0.008 | |
| 36 | Scramlin et al., 2010 | | | Pen | 1 | | | 100 | | 100 | 10 | 547 | | 554 | 1.5 | | 0.95 | | 1.18 | 0.050 | | 8.98 | | 9.07 | 0.120 | | 0.107 | | 0.131 | 0.005 | |
| 83 | Sissom et al 2007 (1) | | | Pen | 1 | | | 274 | | 271 | 51 |  | |  |  | | 1.38 | | 1.42 | 0.010 | | 7.67 | | 7.66 | 0.140 | | 0.179 | | 0.186 | 0.004 | |
| 83 | Sissom et al 2007 (2) | | | Pen | 1 | | | 274 | | 271 | 51 |  | |  |  | | 1.40 | | 1.42 | 0.010 | | 7.79 | | 7.60 | 0.140 | | 0.180 | | 0.187 | 0.004 | |
| 83 | Sissom et al 2007 (3) | | | Pen | 1 | | | 380 | | 387 | 47 |  | |  |  | | 1.60 | | 1.62 | 0.020 | | 8.43 | | 8.47 | 0.090 | | 0.189 | | 0.191 | 0.001 | |
| 83 | Sissom et al 2007 (4) | | | Pen | 1 | | | 377 | | 377 | 47 |  | |  |  | | 1.53 | | 1.58 | 0.020 | | 8.58 | | 8.57 | 0.090 | | 0.179 | | 0.185 | 0.001 | |
| 83 | Sissom et al 2007 (5) | | | Pen | 1 | | | 382 | | 372 | 47 |  | |  |  | | 1.47 | | 1.47 | 0.020 | | 8.55 | | 8.37 | 0.090 | | 0.173 | | 0.178 | 0.001 | |
| 68 | Strydom et al 2011 | | | Individual | | 0 | | 12 | | 12 |  |  | |  |  | |  | |  |  | |  | |  |  | |  | |  |  | |
| 84 | Talton, 2006 | | | Pen | 1 | | | 24 | | 24 | 6 | 582 | | 584 | 3.2 | | 1.27 | | 1.39 | 0.080 | | 11.50 | | 12.40 | 0.440 | | 0.050 | | 0.050 | 0.003 | |
| 85 | Vogel et al., 2009 (1) | | | Pen | 1 | | | 638 | | 628 | 30 | 586 | | 594 | 2.9 | | 1.37 | | 1.61 | 0.060 | | 9.36 | | 9.59 | 0.190 | | 0.145 | | 0.168 | 0.010 | |
| 85 | Vogel et al., 2009 (2) | | | Pen | 1 | | | 638 | | 626 | 30 | 594 | | 593 | 2.9 | | 1.37 | | 1.57 | 0.060 | | 9.36 | | 9.18 | 0.190 | | 0.145 | | 0.169 | 0.010 | |
| 85 | Vogel et al., 2009 (3) | | | Pen | 1 | | | 91 | | 91 | 6 | 606 | | 614 | 5.2 | | 1.50 | | 1.78 | 0.060 | | 10.70 | | 11.18 | 0.060 | | 0.140 | | 0.160 | 0.005 | |
| 86 | Waker et al., 2006 (2) | | Single pens | | 0 | | | 12 | | 12 | 1 | 520 | | 528 | 4.5 | | 1.53 | | 1.80 | 0.160 | | 8.53 | | 8.89 | 0.350 | | 0.178 | | 0.201 | 0.013 | |
| 86 | Walker et al., 2006 (1) | | Single pens | | 0 | | | 12 | | 12 | 1 | 515 | | 535 | 4.5 | | 1.37 | | 2.06 | 0.160 | | 8.55 | | 9.14 | 0.350 | | 0.157 | | 0.222 | 0.013 | |
| 86 | Walker et al., 2006 (3) | | Single pens | | 0 | | | 12 | | 12 | 1 | 528 | | 525 | 4.5 | | 1.81 | | 1.71 | 0.160 | | 9.01 | | 8.29 | 0.350 | | 0.198 | | 0.206 | 0.013 | |
| 87 | Winterholler et al., 2007 | | | Pen | 1 | | | 1126 | | 1126 | 91- 97 | 584 | | 595 | 3.0 | | 1.56 | | 1.63 | 0.010 | | 8.87 | | 8.93 | 0.040 | | 0.176 | | 0.183 | 0.001 | |
| 88 | Winterholler et al., 2008 (1) | | | Pen | 1 | | | 30 | | 30 | 10 | 562 | | 574 | 0.7 | | 0.62 | | 0.94 | 0.090 | | 9.10 | | 10.20 | 0.690 | | 0.070 | | 0.090 | 0.010 | |
| 88 | Winterholler et al., 2008 (2) | | | Pen | 1 | | | 30 | | 30 | 10 | 536 | | 539 | 0.7 | | 0.53 | | 0.67 | 0.090 | | 8.67 | | 7.05 | 0.690 | | 0.060 | | 0.090 | 0.010 | |
| 88 | Winterholler et al., 2008 (3) | | | Pen | 0 | | | 8 | | 8 |  | 590 | | 632 | 13.1 | | 1.36 | | 1.99 | 0.200 | | 8.94 | | 10.20 | 0.780 | | 0.150 | | 0.200 | 0.020 | |
| 88 | Winterholler et al., 2008 (4) | | | Pen | 0 | | | 7 | | 8 |  | 584 | | 547 | 13.1 | | 1.46 | | 0.59 | 0.200 | | 8.58 | | 7.13 | 0.780 | | 0.170 | | 0.090 | 0.020 | |
| 89 | Woerner et al., 2011 (1) | | | Animal | 0 | | | 74 | | 70 |  | 609 | | 604 | 10.1 | | 1.57 | | 1.67 | 0.059 | |  | |  |  | |  | |  |  | |
| 89 | Woerner et al., 2011 (2) | | | Animal | 0 | | | 73 | | 74 |  | 620 | | 630 | 10.1 | | 1.54 | | 1.73 | 0.059 | |  | |  |  | |  | |  |  | |

**Table S8.**Authors**,** \and means for control (Con) and ractopamine treatment (RAC) groups for hot carcass weight (HCW), longissimus muscle area (LMA) and ultimate muscle pH

| Reference | Author | Hot carcass weight  (kg) | | | Longissimus muscle area | | | Ultimate pH | | |
| --- | --- | --- | --- | --- | --- | --- | --- | --- | --- | --- |
|  |  |  |  |  | (cm^2^) | | |  |  |  |
|  |  | Con | RAC | SEM | Con | RAC | SEM | Con | RAC | SEM |
| 70 | Abney et al., 2007 (1) | 359.6 | 362.2 | 7.73 | 86.3 | 89.8 | 1.73 |  |  |  |
| 70 | Abney et al., 2007 (2) | 359.6 | 367.3 | 7.73 | 86.3 | 87.8 | 1.73 |  |  |  |
| 70 | Abney et al., 2007 (3) | 366.9 | 365.7 | 7.73 | 87.6 | 87.3 | 1.73 |  |  |  |
| 70 | Abney et al., 2007 (4) | 366.9 | 373.4 | 7.73 | 87.6 | 91.3 | 1.73 |  |  |  |
| 70 | Abney et al., 2007 (5) | 360.2 | 370.9 | 7.73 | 87.5 | 88.6 | 1.73 |  |  |  |
| 70 | Abney et al., 2007 (6) | 360.2 | 366.9 | 7.73 | 87.5 | 88.9 | 1.73 |  |  |  |
| 71 | Allen et al., 2009 | 416.3 | 414.6 | 15.50 | 75.5 | 82.0 | 6.33 |  |  |  |
| 19 | Avendano-Reyes et al., 2006 | 291.7 | 305.3 | 1.33 | 66.8 | 72.2 | 1.37 | 5.44 | 5.44 | 0.007 |
| 90 | Bass et al., 2009 (1) | 347.6 | 355.1 |  | 69.0 | 71.6 |  |  |  |  |
| 90 | Bass et al., 2009 (2) | 360.4 | 356.0 |  | 72.3 | 74.8 |  |  |  |  |
| 90 | Bass et al., 2009 (3) | 359.3 | 364.6 |  | 75.5 | 78.1 |  |  |  |  |
| 90 | Bass et al., 2009 (4) | 357.3 | 361.2 |  | 74.8 | 74.8 |  |  |  |  |
| 72 | Boler et al., 2012 (1) | 324.6 | 337.8 | 1.24 | 80.4 | 83.9 | 0.98 |  |  |  |
| 72 | Boler et al., 2012 (2) | 324.6 | 339.5 | 1.24 | 80.4 | 84.0 | 0.98 |  |  |  |
| 73 | Bryant et al., 2010 (1) | 362.1 | 363.1 | 5.90 | 84.4 | 84.0 | 1.30 |  |  |  |
| 73 | Bryant et al., 2010 (2) | 362.1 | 368.4 | 5.90 | 84.4 | 86.3 | 1.30 |  |  |  |
| 74 | Glanc, 2013 (1) |  |  |  | 26.9 | 22.60 | 0.93 |  |  |  |
| 74 | Glanc, 2013 (2) |  |  |  | 25.3 | 25.1 | 0.93 |  |  |  |
| 74 | Glanc, 2013 (3) |  |  |  | 23.8 | 25.0 | 0.93 |  |  |  |
| 74 | Glanc, 2013 (4) |  |  |  | 24.4 | 25.5 | 0.93 |  |  |  |
| 74 | Glanc, 2013 (5) |  |  |  | 25.4 | 28.8 | 0.93 |  |  |  |
| 74 | Glanc, 2013 (6) |  |  |  | 24.5 | 23.3 | 0.93 |  |  |  |
| 91 | Griffin et al., 2009a | 405.0 | 405.0 | 1.00 | 91.0 | 91.0 | 0.77 |  |  |  |
| 92 | Gruber et al., 2007 | 359.3 | 364.8 | 4.90 | 81.7 | 84.0 | 1.10 |  |  |  |
| 61 | Holmer et al., 2009c (1) | 278.0 | 356.9 | 9.94 | 68.8 | 80.4 | 1.79 | 5.62 | 5.52 | 0.037 |
| 61 | Holmer et al., 2009c (1) | 352.8 | 356.9 | 9.94 | 78.2 | 80.4 | 1.79 | 5.53 | 5.52 | 0.037 |
| 77 | Jennings, 2011 | 327.7 | 333.3 | 1.63 | 85.9 | 88.2 | 0.38 |  |  |  |
| 78 | Laudert et al., 2004 (1) | 374.7 | 377.1 | 1.68 | 86.3 | 86.9 | 1.69 |  |  |  |
| 78 | Laudert et al., 2004 (2) | 374.7 | 380.3 | 1.68 | 86.3 | 88.1 | 1.69 |  |  |  |
| 30 | Peterson, 2011 | 375.3 | 378.7 | 4.00 | 83.5 | 85.2 | 0.90 |  |  |  |
| 79 | Quinn et al., 2008 Exp 1 (1) | 334.0 | 335.0 | 2.50 | 86.3 | 88.2 | 0.20 |  |  |  |
| 79 | Quinn et al., 2008 Exp 2 (2) | 304.0 | 308.0 | 2.50 | 83.9 | 83.2 | 0.20 |  |  |  |
| 79 | Quinn et al., 2008 Exp 2 (3) | 304.0 | 307.0 | 2.50 | 83.9 | 83.2 | 0.20 |  |  |  |
| 79 | Quinn et al., 2008 Exp 2 (4) | 304.0 | 312.0 | 2.50 | 83.9 | 83.9 | 0.20 |  |  |  |
| 80 | Schluter (1991) (1) | 302.0 | 305.0 | 2.11 | 78.8 | 79.2 | 1.03 |  |  |  |
| 80 | Schluter (1991) (2) | 302.0 | 310.0 | 2.11 | 78.8 | 79.3 | 1.03 |  |  |  |
| 80 | Schluter (1991) (3) | 302.0 | 310.0 | 2.11 | 78.8 | 81.0 | 1.03 |  |  |  |
| 81 | Schroeder et al., 2003 (1) | 342.0 | 344.6 | 13.17 | 75.0 | 76.9 | 2.69 |  |  |  |
| 81 | Schroeder et al., 2003 (2) | 342.0 | 348.4 | 13.17 | 75.0 | 77.5 | 2.69 |  |  |  |
| 81 | Schroeder et al., 2003 (3) | 342.0 | 350.3 | 13.17 | 75.0 | 78.1 | 2.69 |  |  |  |
| 82 | Schroeder et al., 2005 (1) | 315.5 | 316.3 | 6.50 | 81.3 | 81.9 | 2.77 |  |  |  |
| 82 | Schroeder et al., 2005 (2) | 315.5 | 318.3 | 6.50 | 81.3 | 81.9 | 2.77 |  |  |  |
| 82 | Schroeder et al., 2005 (3) | 315.5 | 320.6 | 6.50 | 81.3 | 84.5 | 2.77 |  |  |  |
| 36 | Scramlin et al., 2010 | 357.8 | 363.1 | 1.36 | 34.2 | 34.2 | 0.43 | 5.46 | 5.45 | 0.010 |
| 83 | Sissom et al 2007 (1) | 343.0 | 349.0 | 2.00 | 93.3 | 95.7 | 0.80 |  |  |  |
| 83 | Sissom et al 2007 (2) | 346.0 | 350.0 | 2.00 | 94.2 | 96.9 | 0.80 |  |  |  |
| 83 | Sissom et al 2007 (3) | 313.0 | 315.0 | 2.00 | 91.2 | 93.5 | 0.10 |  |  |  |
| 83 | Sissom et al 2007 (4) | 329.0 | 335.0 | 2.00 | 94.2 | 96.1 | 0.10 |  |  |  |
| 83 | Sissom et al 2007 (5) | 348.0 | 347.0 | 2.00 | 95.5 | 94.2 | 0.10 |  |  |  |
| 68 | Strydom et al 2011 | 339.1 | 346.1 | 2.11 | 75.5 | 80.0 | 2.65 |  |  |  |
| 84 | Talton, 2006 | 359.7 | 367.8 | 2.47 | 79.4 | 84.1 | 1.42 | 5.50 | 5.48 | 0.020 |
| 85 | Vogel et al., 2009 (1) | 357.5 | 362.2 | 1.43 | 77.0 | 78.8 | 1.61 |  |  |  |
| 85 | Vogel et al., 2009 (2) | 357.5 | 352.6 | 1.43 | 77.0 | 79.8 | 1.61 |  |  |  |
| 85 | Vogel et al., 2009 (3) | 346.7 | 352.2 | 2.61 | 71.9 | 73.6 | 0.98 |  |  |  |
| 86 | Waker et al., 2006 (2) | 316.4 | 323.7 | 3.20 | 83.5 | 90.4 | 3.50 |  |  |  |
| 86 | Walker et al., 2006 (1) | 318.0 | 327.8 | 3.20 | 91.5 | 86.1 | 3.50 |  |  |  |
| 86 | Walker et al., 2006 (3) | 319.3 | 322.9 | 3.20 | 81.6 | 91.3 | 3.50 |  |  |  |
| 87 | Winterholler et al., 2007 | 371.0 | 379.0 | 2.00 | 90.7 | 92.4 | 0.66 |  |  |  |
| 88 | Winterholler et al., 2008 (1) | 358.0 | 368.0 | 5.00 | 85.3 | 93.2 | 2.52 |  |  |  |
| 88 | Winterholler et al., 2008 (2) | 341.0 | 341.0 | 5.00 | 84.8 | 86.6 | 2.52 |  |  |  |
| 88 | Winterholler et al., 2008 (3) | 374.0 | 395.0 | 8.69 | 97.9 | 103.1 | 7.29 |  |  |  |
| 88 | Winterholler et al., 2008 (4) | 365.0 | 339.0 | 8.69 | 99.9 | 83.0 | 7.29 |  |  |  |
| 89 | Woerner et al., 2011 (1) | 370.0 | 371.0 | 5.30 | 88.1 | 88.4 | 1.07 |  |  |  |
| 89 | Woerner et al., 2011 (2) | 381.0 | 387.0 | 5.30 | 91.2 | 94.4 | 1.07 |  |  |  |

*Glanc removed from LMA weighted mean difference estimation

**Table S9.**Authors and means for control (Con) and ractopaminetreatment (RAC) groups for USDA marbling score , fat thickness (mm) and dressing percentage (D%)

| Reference | Author |  | USDA Marbling score | | |  |  | Fat thickness (mm) | | |  |  | D% | | |  |
| --- | --- | --- | --- | --- | --- | --- | --- | --- | --- | --- | --- | --- | --- | --- | --- | --- |
|  |  | Con | | RAC | SEM | | Control | | RAC | SEM | | Con | | RAC | SEM | |
| 70 | Abney et al., 2007 (1) | 381 | | 391 | 11.3 | | 12.0 | | 11.5 | 0.70 | | 61.4 | | 62.0 | 0.330 | |
| 70 | Abney et al., 2007 (2) | 381 | | 409 | 11.3 | | 12.0 | | 12.4 | 0.70 | | 61.4 | | 62.1 | 0.330 | |
| 70 | Abney et al., 2007 (3) | 395 | | 401 | 11.3 | | 12.3 | | 11.8 | 0.70 | | 62.0 | | 61.8 | 0.330 | |
| 70 | Abney et al., 2007 (4) | 395 | | 390 | 11.3 | | 12.3 | | 11.9 | 0.70 | | 62.0 | | 62.1 | 0.330 | |
| 70 | Abney et al., 2007 (5) | 394 | | 411 | 11.3 | | 12.3 | | 12.1 | 0.70 | | 62.1 | | 61.9 | 0.330 | |
| 70 | Abney et al., 2007 (6) | 394 | | 399 | 11.3 | | 12.3 | | 12.1 | 0.70 | | 62.1 | | 61.8 | 0.330 | |
| 71 | Allen et al., 2009 | 539 | | 522 | 60.0 | | 7.4 | | 8.5 | 1.33 | | 54.8 | | 54.6 | 1.130 | |
| 19 | Avendano-Reyes et al., 2006 |  | |  |  | | 16.5 | | 15.6 | 0.58 | | 61.0 | | 62.5 | 0.217 | |
| 90 | Bass et al., 2009 (1) | 580 | | 619 |  | | 7.6 | | 7.2 |  | |  | |  |  | |
| 90 | Bass et al., 2009 (2) | 542 | | 543 |  | | 6.9 | | 6.7 |  | |  | |  |  | |
| 90 | Bass et al., 2009 (3) | 521 | | 519 |  | | 6.3 | | 6.5 |  | |  | |  |  | |
| 90 | Bass et al., 2009 (4) | 565 | | 535 |  | | 6.8 | | 6.8 |  | |  | |  |  | |
| 72 | Boler et al., 2012 (1) | 471 | | 454 | 14.6 | | 13.5 | | 13.5 | 0.50 | | 62.3 | | 63.1 | 0.30 | |
| 72 | Boler et al., 2012 (2) | 471 | | 444 | 14.6 | | 13.5 | | 14.0 | 0.50 | | 62.3 | | 63.4 | 0.30 | |
| 73 | Bryant et al., 2010 (1) | 535 | | 538 | 8.2 | | 15.2 | | 14.7 | 0.34 | | 63.1 | | 62.9 | 0.29 | |
| 73 | Bryant et al., 2010 (2) | 535 | | 531 | 8.3 | | 15.2 | | 14.8 | 0.34 | | 63.1 | | 63.6 | 0.29 | |
| 73 | Bryant et al., 2010 (3) | 582 | | 548 | 26.0 | | 11.2 | | 12.4 | 0.60 | | 65.4 | | 65.4 | 0.30 | |
| 74 | Glanc, 2013 (1) | 420 | | 500 | 26.4 | |  | |  |  | |  | |  |  | |
| 74 | Glanc, 2013 (2) | 517 | | 407 | 26.4 | |  | |  |  | |  | |  |  | |
| 74 | Glanc, 2013 (3) | 456 | | 410 | 26.4 | |  | |  |  | |  | |  |  | |
| 74 | Glanc, 2013 (4) | 453 | | 437 | 26.4 | |  | |  |  | |  | |  |  | |
| 74 | Glanc, 2013 (5) | 415 | | 428 | 26.4 | |  | |  |  | |  | |  |  | |
| 74 | Glanc, 2013 (6) | 462 | | 455 | 26.4 | |  | |  |  | |  | |  |  | |
| 91 | Griffin et al., 2009a | 575 | | 574 | 32.2 | | 11.9 | | 11.9 | 0.10 | |  | |  |  | |
| 7 | Gruber et al., 2007 | 487 | | 477 | 5.2 | | 13.2 | | 12.9 | 0.02 | | 64.0 | | 64.2 | 0.26 | |
| 74 | Holmer et al., 2009c (1) | 438 | | 551 | 26.8 | | 4.0 | | 13.0 | 1.8 | | 55.6 | | 58.4 | 1.03 | |
| 74 | Holmer et al., 2009c(2) | 547 | | 551 | 26.8 | | 12.0 | | 13.0 | 1.8 | | 57.2 | | 58.4 | 1.03 | |
| 77 | Jennings, 2011 | 425 | | 407 | 7.8 | | 14.5 | | 13.50 | 0.50 | | 62.4 | | 62.7 | 0.00 | |
| 78 | Laudert et al., 2004 (1) | 504 | | 504 | 6.4 | | 12.8 | | 12.5 | 0.100 | | 63.9 | | 63.9 | 0.240 | |
| 78 | Laudert et al., 2004 (2) | 504 | | 501 | 6.4 | | 12.8 | | 12.75 | 1.00 | | 63.9 | | 64.1 | 0.24 | |
| 31 | Peterson, 2011 | 431 | | 411 | 5.5 | | 12.7 | | 1.190 | 0.30 | | 63.4 | | 63.2 | 0.44 | |
| 79 | Quinn et al., 2008 Exp 1 (1) | 380 | | 373 | 7.0 | | 7.9 | | 7.6 | 0.3 | |  | |  |  | |
| 79 | Quinn et al., 2008 Exp 2 (2) | 438 | | 431 | 13.0 | | 9.1 | | 9.9 | 0.02 | | 63.800 | | 63.900 | 0.500 | |
| 79 | Quinn et al., 2008 Exp 2 (3) | 438 | | 411 | 13.0 | | 9.1 | | 10.2 | 0.02 | | 63.800 | | 64.500 | 0.500 | |
| 79 | Quinn et al., 2008 Exp 2 (4) | 438 | | 427 | 13.0 | | 9.1 | | 10.9 | 0.02 | | 63.800 | | 65.200 | 0.500 | |
| 80 | Schluter (1991) (1) |  | |  |  | | 9.3 | | 0.970 | 4.70 | | 61.3 | | 61.4 | 0.25 | |
| 80 | Schluter (1991) (2) |  | |  |  | | 9.3 | | 0.940 | 4.70 | | 61.3 | | 61.5 | 0.25 | |
| 80 | Schluter (1991) (3) |  | |  |  | | 9.3 | | 0.990 | 4.70 | | 61.3 | | 61.4 | 0.25 | |
| 81 | Schroeder et al., 2003 (1) | 533 | | 528 | 18.2 | | 14.3 | | 14.00 | 1.25 | |  | |  |  | |
| 81 | Schroeder et al., 2003 (2) | 533 | | 531 | 18.2 | | 14.3 | | 14.00 | 1.25 | |  | |  |  | |
| 81 | Schroeder et al., 2003 (3) | 533 | | 525 | 18.2 | | 14.3 | | 14.00 | 1.25 | |  | |  |  | |
| 82 | Schroeder et al., 2005 (1) |  | |  |  | | 16.0 | | 16.00 | 1.80 | | 62.2 | | 62.0 | 0.70 | |
| 82 | Schroeder et al., 2005 (2) |  | |  |  | | 16.0 | | 16.00 | 1.80 | | 62.2 | | 62.0 | 0.70 | |
| 82 | Schroeder et al., 2005 (3) |  | |  |  | | 16.0 | | 15.70 | 1.80 | | 62.2 | | 62.1 | 0.70 | |
| 36 | Scramlin et al., 2010 | 546 | | 533 | 8.7 | | 12.4 | | 12.2 | 0.05 | | 65.5 | | 65.5 | 0.180 | |
| 83 | Sissom et al 2007 (1) | 425 | | 409 | 8.0 | | 12.4 | | 11.4 | 0.03 | |  | |  |  | |
| 83 | Sissom et al 2007 (2) | 406 | | 398 | 8.0 | | 12.4 | | 11.7 | 0.03 | |  | |  |  | |
| 83 | Sissom et al 2007 (3) | 477 | | 472 | 6.0 | | 11.9 | | 12.2 | 0.03 | |  | |  |  | |
| 83 | Sissom et al 2007 (4) | 484 | | 478 | 6.0 | | 14.5 | | 13.5 | 0.03 | |  | |  |  | |
| 83 | Sissom et al 2007 (5) | 501 | | 506 | 6.0 | | 15.2 | | 15.2 | 0.03 | |  | |  |  | |
| 37 | Strydom et al (2009) | 221 | | 234 | 9.9 | | 14.2 | | 12.70 | 1.42 | | 59.6 | | 59.4 | 0.29 | |
| 84 | Talton, 2006 | 426 | | 434 | 28.6 | | 15.0 | | 1.570 | 1.00 | | 61.8 | | 63.0 | 0.14 | |
| 85 | Vogel et al., 2009 (1) | 515 | | 498 | 20.7 | | 6.6 | | 6.4 | 0.03 | | 61.2 | | 61.2 | 0.510 | |
| 85 | Vogel et al., 2009 (2) | 515 | | 507 | 20.7 | | 6.6 | | 5.8 | 0.03 | | 61.2 | | 61.4 | 0.510 | |
| 85 | Vogel et al., 2009 (3) | 556 | | 549 | 12.6 | | 5.2 | | 4.8 | 0.03 | | 58.3 | | 59.1 | 0.010 | |
| 86 | Waker et al., 2006 (2) | 390 | | 338 | 22.0 | | 6.6 | | 8.5 | 0.09 | | 60.9 | | 61.5 | 0.530 | |
| 86 | Walker et al., 2006 (1) | 338 | | 344 | 22.0 | | 6.9 | | 7.4 | 0.09 | | 61.7 | | 61.4 | 0.530 | |
| 86 | Walker et al., 2006 (3) | 346 | | 348 | 22.0 | | 7.6 | | 7.6 | 0.09 | | 60.5 | | 61.5 | 0.530 | |
| 87 | Winterholler et al., 2007 | 492 | | 496 | 3.0 | | 14.7 | | 14.9 | 0.03 | | 63.3 | | 63.6 | 0.140 | |
| 88 | Winterholler et al., 2008 (1) | 383 | | 349 | 9.0 | | 10.7 | | 9.1 | 0.05 | | 63.7 | | 64.1 | 0.400 | |
| 88 | Winterholler et al., 2008 (2) | 358 | | 342 | 9.0 | | 8.4 | | 8.6 | 0.05 | | 63.7 | | 63.3 | 0.400 | |
| 88 | Winterholler et al., 2008 (3) | 362 | | 410 | 31.8 | | 10.7 | | 11.3 | 0.21 | | 63.4 | | 62.5 | 0.880 | |
| 88 | Winterholler et al., 2008 (4) | 395 | | 458 | 31.8 | | 10.2 | | 12.4 | 0.21 | | 62.5 | | 61.8 | 0.880 | |
| 89 | Woerner et al., 2011 (1) | 438 | | 429 | 10.5 | |  | |  |  | |  | |  |  | |
| 89 | Woerner et al., 2011 (2) | 418 | | 404 | 10.5 | |  | |  |  | |  | |  |  | |

**Table S10.**Authors**,** experimental unit, days aged, number of animals, number of pens, number of muscles and means for control (Con) and ractopamine treatment (RAC) groups for Warner –Bratzler shear force and cooking loss

| References | Author | Experimental unit | | Age | No of animals | | No of pens | | No of muscles | | Warner –Bratzler shear force (kg) | | | Cooking loss (%) | | |
| --- | --- | --- | --- | --- | --- | --- | --- | --- | --- | --- | --- | --- | --- | --- | --- | --- |
|  |  | |  |  | Con | RAC | Con | RAC | Con | RAC | Con | RAC | SE | Con | RAC | SE |
| 71 | Allen et al., 2009 | | Animal |  | 8 | 9 |  |  |  |  | 4.32 | 4.51 | 0.347 |  |  |  |
| 19 | Avendano-Reyes et al., 2006 | | Pen |  | 6 | 6 |  |  |  |  | 4.40 | 4.83 | 0.136 |  |  |  |
| 72 | Boler et al., 2012 | | Strip loin |  | 6 | 6 |  |  |  |  | 2.28 | 2.42 | 0.090 |  |  |  |
| 72 | Boler et al., 2012 | | Strip loin |  | 6 | 6 |  |  |  |  | 2.28 | 2.47 | 0.090 |  |  |  |
| 74 | Glanc, 2013 | | Animal | 14 | 1 | 1 |  |  |  |  | 3.94 | 4.13 | 0.069 | 22.4 | 23 | 0.22 |
| 92 | Gruber et al., 2008 | | Pen |  | 21 | 21 |  |  |  |  | 4.22 | 4.22 | 0.060 |  |  |  |
| 77 | Jennings, 2011 | | Pen |  | 9 | 9 |  |  |  |  | 5.65 | 5.62 | 0.200 |  |  |  |
| 77 | Jennings, 2011 | | Pen |  | 9 | 9 |  |  |  |  | 4.79 | 4.9 | 0.140 |  |  |  |
| 77 | Jennings, 2011 | | Pen |  | 9 | 9 |  |  |  |  | 3.67 | 4.12 | 0.120 |  |  |  |
| 77 | Jennings, 2011 | | Pen |  | 9 | 9 |  |  |  |  | 3.63 | 3.62 | 0.100 |  |  |  |
| 79 | Quinn et al., 2008 | | Pen | 14 | 22 | 22 |  |  |  |  | 4.60 | 4.60 | 0.500 | 25 | 25 | 14 |
| 36 | Scramlin et al., 2010 | | Strip loin | 3 | 10 | 10 |  |  |  |  | 4.66 | 5.36 | 0.200 |  |  |  |
| 36 | Scramlin et al., 2010 | | Strip loin | 7 | 10 | 10 |  |  |  |  | 4.15 | 4.63 | 0.200 |  |  |  |
| 36 | Scramlin et al., 2010 | | Strip loin | 14 | 10 | 10 |  |  |  |  | 3.55 | 3.78 | 0.200 |  |  |  |
| 36 | Scramlin et al., 2010 | | Strip loin | 21 | 10 | 10 |  |  |  |  | 3.05 | 3.28 | 0.200 |  |  |  |
| 37 | Strydom et al (2009) | | Animal |  | 12 | 12 |  |  |  |  | 4.60 | 5.00 | 0.109 |  |  |  |
| 84 | Talton, 2006 | | Animal |  |  |  |  |  |  |  |  |  |  | 20.3 | 19.8 | 0.41 |
| 89 | Woerner et al., 2011 | | Animal |  | 74 | 70 |  |  |  |  | 3.48 | 3.72 | 0.062 |  |  |  |
| 89 | Woerner et al., 2011 | | Animal |  | 73 | 74 |  |  |  |  | 3.60 | 3.83 | 0.062 |  |  |  |

**Table S11.**List of ractopaminestudies that did not meet the selection criteria, insufficient data or data provided were not appropriate

| Reference | Studies | Reasons for exclusion |
| --- | --- | --- |
| 93 | Beerman 2002 | Review |
| 94 | Dijkhuis et al., 2008 | Data were not appropriate |
| 95 | Eisemann and Bristol | Data were not appropriate |
| 96 | Gonzales et al., 2007 | Data were not appropriate |
| 97 | Gonzales et al., 2008 | Data were not appropriate |
| 98 | Gonzales et al., 2009 | Data were not appropriate |
| 99 | Griffin et al., 2009b | Data were not appropriate |
| 100 | Li and Jiang 2012 | Topic not relevant |
| 101 | Paddock et al 2011 | Topic not relevant |
| 102 | Shelver and Smith 2002 | Data were not appropriate |
